# A STRUBBELIG–NHL3 cell surface receptor complex mediates the response to cellulose deficiency in *Arabidopsis*

**DOI:** 10.1038/s41477-026-02342-4

**Published:** 2026-08-14

**Authors:** Rodion Boikine, Ajeet Chaudhary, Julia Mergner, Henriette Leicher, Martin Stegmann, Bernhard Kuster, Kay Schneitz

**Affiliations:** 1 Plant Developmental Biology, TUM School of Life Sciences, Technical University of Munich, Freising, Germany; 2 Chair of Proteomics and Bioanalytics, TUM School of Life Sciences, Technical University of Munich, Freising, Germany; 3 Bavarian Center for Biomolecular Mass Spectrometry Center (BayBioMS), TUM School of Life Sciences, Technical University of Munich, Freising, Germany; 4 Chair of Phytopathology, TUM School of Life Sciences, Technical University of Munich, Freising, Germany; 5 Molecular Botany, Institute of Botany, Ulm University, Ulm, Germany; 6 Present Address: Department of Plant Biology, Carnegie Institution for Science, Stanford, CA USA

**Keywords:** Plant signalling, Abiotic

## Abstract

Plant cells are enclosed by a semi-rigid cell wall with a complex biochemical composition and architecture. The poorly understood process of remodelling the cell wall is crucial for controlling growth and development and for regulating abiotic and biotic stress responses. Cell wall remodelling upon disruption of cell wall integrity through inhibition of cellulose biosynthesis depends on the receptor kinase STRUBBELIG (SUB) and its binding partner QUIRKY (QKY). Here, we identify NON-RACE SPECIFIC DISEASE RESISTANCE/HIN1 HAIRPIN-INDUCED-LIKE protein 3 (NHL3) as an additional factor involved in the SUB-dependent cellulose biosynthesis inhibition response. Collectively, our data indicate that NHL3 maintains SUB at the plasma membrane by physically interacting with SUB. SUB signalling is attenuated by receptor-mediated endocytosis initiated by release of first the SUB–NHL3 and later the SUB–QKY interaction. Our results further suggest a diverse set of biochemically and functionally distinct SUB complexes involved in regulating cell wall integrity and development.

## Abstract (web summary)

This study shows that signalling by the multifunctional receptor kinase SUB in cell wall integrity involves two functionally distinct cell surface complexes whose activities are attenuated by sequential endocytosis, revealing a stress–growth trade-off.

## Main

Plant cells are encased by a semi-rigid cell wall that resists turgor pressure, physically connects plant cells, modulates cell growth and is an important component of the abiotic and biotic stress response^1^. A plethora of developmental and stress-related cues induce a multitude of changes in the cell wall. Thus, monitoring and maintaining cell wall integrity is of central importance for plant development and survival. However, the molecular framework underlying signalling processes in response to cell wall cues remains poorly understood^2–5^.

Cellulose microfibrils constitute central load-bearing elements of the cell wall^6,7^. Cellulose biosynthesis inhibition (CBI), caused by genetic perturbation or the application of inhibitors such as isoxaben and thaxtomin A, elicits a compensatory response in plant cells, including changes in their transcriptional and hormonal status and remodelling of the cell wall through the accumulation of lignin in seedling roots or callose in cotyledons^8–12^. In addition, CBI causes developmental defects, including aberrant root hair patterning and defective ovule morphogenesis^13^.

Several cell surface receptor kinases (RKs) have been identified as components of a complex signalling network that mediates the seedling response to CBI, including THESEUS 1 (THE1), MALE DISCOVERER 1-INTERACTING RECEPTOR-LIKE KINASE 2 (MIK2; also known as LEUCINE-RICH REPEAT KINASE FAMILY PROTEIN INDUCED BY SALT STRESS (LRR-KISS)) and STRUBBELIG (SUB (At1G11130); also known as SCRAMBLED (SCM))^11,13–18^. Upon application of isoxaben, THE1 promotes the expression of several ligands of MIK2, which in turn activate MIK2 (ref. ^19^). SUB is a multifunctional RK that is known to mediate CBI responses, including lignification of the seedling root tip and accumulation of callose in cotyledons^17^. SUB also controls developmental processes, including root hair patterning and ovule morphogenesis^13,20,21^. Interestingly, CBI-induced defects in these developmental processes are caused by downregulation of SUB activity^13^.

SUB controls CBI responses independently of THE1 and MIK2 (ref. ^17^). QUIRKY (QKY) (At1G74720) is the only other factor known to be involved in the SUB-dependent CBI response. QKY promotes CBI-induced lignification in the seedling root tip but does not function in callose accumulation in cotyledons^17^. QKY is also required for SUB-mediated developmental processes^22,23^. It is an endoplasmic reticulum-anchored protein containing multiple C2 domains and two transmembrane regions, and is present at plasmodesmata^22,24–27^. At plasmodesmata and possibly other endoplasmic reticulum–plasma membrane contact sites, the intracellular domain of SUB physically interacts with a region that includes the amino-terminal (N-terminal) C2 domains of QKY to promote SUB signalling^24,27^. Ligand-induced, receptor-mediated endocytosis is a common element in the spatiotemporal regulation of RK signalling^28,29^. Some plant RKs, including BRASSINOSTEROID INSENSITIVE 1 (BRI1), ARABIDOPSIS CRINKLY 4 (ACR4) and SUB, are constantly internalized^30–32^. The physical interaction between SUB and QKY maintains SUB at the cell surface by attenuating its constitutive internalization^13,27,33^. Supporting this model, *SUB* overexpression rescues *qky* mutant phenotypes^24^.

Here, we report the identification of NON-RACE SPECIFIC DISEASE RESISTANCE/HIN1 HAIRPIN-INDUCED-LIKE protein 3 (NHL3) as a new physical interactor of SUB involved in SUB-dependent cell wall remodelling during the CBI response.

## Results

### NHL3 physically interacts with SUB

**Fig. 1:**
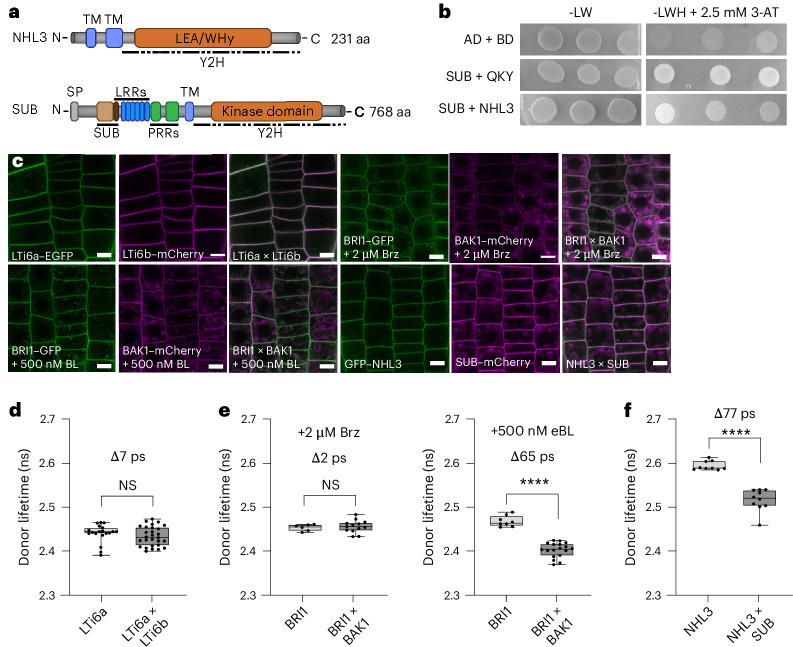
NHL3 physically interacts with SUB. **a**, Cartoon showing the domain structures of the NHL3 and SUB proteins. Regions used for Y2H assays are indicated. **b**, Y2H assay with SUB fused to the GAL4 activation domain (AD) and NHL3 and QKY fused to the GAL4 DNA binding domain (BD). SUB and the C2A-D domain of QKY are used as positive controls. Growth on double drop-out media (-LW) indicates successful transformation of both plasmids, and on triple drop-out media (-LWH) indicates the presence of an interaction. **c**, Confocal micrographs of *A. thaliana* expressing the indicated constructs and the merged image of epidermal cell files in the root meristem. **d**, Quantification of the fluorescence lifetime of LTi6a–GFP in the absence and presence of LTi6b–2mCherry at the cell surface. The reduction in donor fluorescence lifetime indicates physical interaction of proteins. Box-and-whisker plots show the median (horizontal line), the 25th–75th percentiles (box) and the 5th–95th percentiles (whiskers). Data are shown as mean ± s.d. for LTi6a (*n* = 19 individual seedlings and LTi6a × LTi6b (*n* = 28 individual seedlings), with each data point representing the average of at least 5 cells per root. **e**, Left: quantification of the fluorescence lifetime of BRI1–GFP in the absence and presence of BAK1–mCherry after treatment with 2 μM brassinazole (Brz) at the plasma membrane. Right: quantification of the fluorescence lifetime of BRI1–GFP in the absence and presence of BAK1–mCherry after treatment with 500 nM epibrassinolide (eBL) at the plasma membrane. The reduction in lifetime indicates physical interaction of proteins. Left: data are shown as mean ± s.d. for BRI1 (*n* = 6 individual seedlings) and BRI1 × BAK1 (*n* = 13 individual seedlings), with each data point representing the average of at least 5 cells per root. Right: data are shown as mean ± s.d. for BRI1 (*n* = 8 individual seedlings) and BRI1 × BAK1 (*n* = 18 individual seedlings), with each data point representing the average of at least 5 cells per root. **f**, Quantification of the fluorescence lifetime of GFP–NHL3 in the absence and presence of SUB–mCherry at the cell surface. Left: data are shown as mean ± s.d. for NHL3 (*n* = 9 individual seedlings) and NHL3 × SUB (*n* = 10 individual seedlings), with each data point representing the average of at least 5 cells per root. Statistical parameters and exact *P* values are shown in the Source data. Similar results were obtained from at least two (**d**) or three (**e**,**f**) independent experiments. Scale bars, 8 μm. ****P < 0.0001, unpaired *t* test, two-tailed *P* values. NS, not significant. Source data

**Extended Data Fig. 1:**
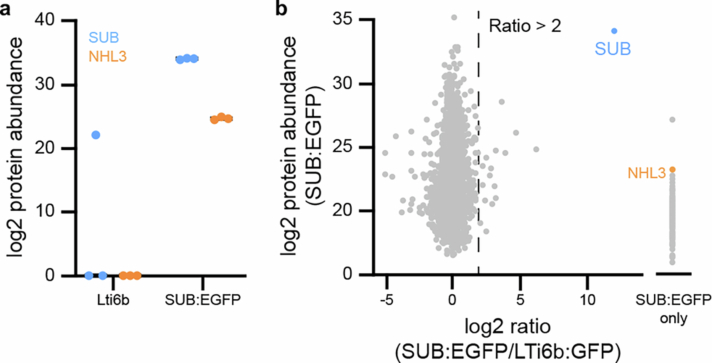
Co-immunoprecipitation (co-IP) experiment of 7-d-old wild-type seedlings expressing SUB:EGFP. A pUBQ::LTi6b:GFP line served as negative control. Proteins bound to anti-GFP nanobodies coupled to agarose beads were analyzed by bottom-up liquid chromatography-mass spectrometry (LC-MS). **a**, Protein abundance of SUB:GFP (SUB) and NHL3 from GFP-trap co-immunoprecipitation in triplicates. **b**, Ratio between the average protein abundance in SUB:EGFP versus the negative control LTi6b:GFP plotted against the protein abundance in SUB:EGFP. NHL3 is only detected in SUB:EGFP. Displayed are proteins with at least two quantitative identifications in the SUB:EGFP IP samples. Source data

To identify components of SUB complexes, we performed a co-immunoprecipitation (co-IP) experiment using 7-day-old wild-type seedlings carrying a functional transgene encoding a SUB–EGFP translational fusion driven by its endogenous promoter (pSUB::SUB:EGFP)^24^. As a negative control, we used a line transgenic for a translational fusion between *Arabidopsis* LOW TEMPERATURE-INDUCED PROTEIN 6 (LTi6) and GFP (pUBQ::LTi6b:GFP*)*. Proteins bound to anti-GFP nanobodies coupled to agarose beads were analysed by mass spectrometry. Candidates specific for complex formation with SUB–EGFP were further analysed. NHL3 (AT5G06320) was robustly detected in three independent biological replicates (Extended Data Fig. 1). NHL3 carries two predicted transmembrane domains towards the N terminus, followed by a late embryogenesis abundant (LEA) domain of subgroup 2, also described as a water stress and hypersensitive response (WHy) domain^34^ (Fig. 1a). LEA domain proteins are considered intrinsically disordered in solution and have been suggested to play important roles in stress responses by acting as chaperones and protectors of protein and membrane structures^35,36^. NHL3 is known to be involved in pattern-triggered plant immunity^37–39^ and to participate in callose deposition and plasmodesmata closure induced by multiple immune signals^40^.

**Extended Data Fig. 2:**
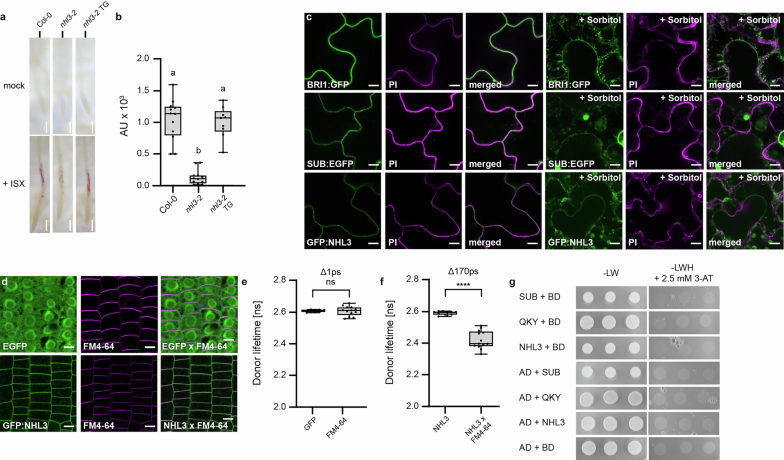
Functionality of the pUBQ::GFP:NHL3 (nhl3-2 TG) reporter, NHL3 plasma membrane localization, and NHL3 non self-autoactivation in yeast. **a**, Phloroglucinol signal intensity indicating lignin accumulation in roots of 7 d old Col-0 and mutant seedlings exposed to 600 nM isoxaben for 16 h. Genotypes are indicated. **b**, Quantification of the results shown in a. Box-and-whisker plots show the median (horizontal line), the 25th–75th percentiles (box), and the 5th–95th percentiles (whiskers). Data are shown as mean ± s.d. for Col-0 (n = 12 individual seedlings), nhl3-2 (n = 12 individual seedlings) and nhl3-2TG (n = 9 individual seedlings) with each data point representing one root. Columns not sharing a letter are significantly different in a one-way ANOVA followed by Tukey’s multiple comparison test (P < 0.05). Scale bars: 100 μm. **c**, Cotyledon pavement cells of 7 d old *A. thaliana* seedlings before and after treatment with 1 M sorbitol for 30 min. Reporter constructs are indicated. The cell wall was counterstained with 5 μg/ml propidium iodide (PI) for 5 min before mounting for observation. Note successfully plasmolysed apoplast in all samples. The images are representative of n = 12 for BRI1:GFP, n = 10 for SUB:EGFP (pUBQ::SUB:EGFP) and n = 20 for GFP:NHL3 individual seedlings. Scale bars: 8 μm. **d**, Confocal micrographs of A. thaliana expressing EGFP and GFP:NHL3 constructs in the presence and absence of the PM-labelled FM4-64 stain and the merged image in epidermal cell files in the root meristem. Scale bars: 8 μm. **e**, Quantification of GFP fluorescence lifetime in the absence and presence of 1 μM FM4-64 at the PM. Reduction in lifetime correlates with direct physical interaction. Box-and-whisker plots show the median (horizontal line), the 25th–75th percentiles (box), and the 5th–95th percentiles (whiskers). Data are shown as mean ± s.d. for GFP (n = 9 individual seedlings) and GFP x FM4-64 (n = 13 individual seedlings). Each data point represents the average of at least 5 cells per root. ns, not significant; unpaired t test, two-tailed P values. **f**, Quantification of the fluorescence lifetime of NHL3 in the absence and presence of 1 μM FM4-64 at the PM. Reduction in lifetime correlates with direct physical interaction. Box-and-whisker plots show the median (horizontal line), the 25th–75th percentiles (box), and the 5th–95th percentiles (whiskers). Data are shown as mean ± s.d. for NHL3 (n = 10 individual seedlings) and NHL3 x FM4-64 (n = 13 individual seedlings). Each data point represents the average of at least 5 cells per root. ns, not significant; unpaired t test, two-tailed P values (****P < 0.0001). **g**, Transcription autoactivation analysis of a yeast two-hybrid assay with SUB, QKY and NHL3 fused to the GAL4 activation domain (AD) and to the GAL4 DNA binding domain (BD), respectively. Growth on double drop-out media (-LW) indicates successful transformation of both plasmids. No growth on triple drop-out media (-LWH) indicates the absence of self-autoactivation. Similar results were obtained in one (**c**) two (**b**,**e**) or three (**f**) independent experiments. Statistical parameters and exact P values are shown in the Source Data file. Scale bars: 8 μm. Source data

A search of public databases revealed that *NHL3* and *SUB* show largely overlapping spatial expression patterns in seedlings, for example in the root tip and in cotyledons, supporting a possible interaction between the proteins (eFP browser: https://bar.utoronto.ca/eplant/? (ref. ^41^)). SUB localizes to the plasma membrane^42^. We observed plasma membrane localization of a functional GFP–NHL3 reporter (Extended Data Fig. 2), driven by the *UBIQUITIN 10* promoter, in plasmolysis experiments in cotyledon pavement cells. This was confirmed using steady-state Förster resonance energy transfer measured via fluorescence lifetime microscopy (FRET-FLIM) with the plasma membrane stain FM4-64 in epidermal cells of the root meristem (Extended Data Fig. 2c–e), consistent with previous results^37,40^.

To assess whether SUB can physically interact with NHL3, we performed yeast two-hybrid (Y2H) assays using the intracellular domain of SUB and the carboxy-terminal intracellular domain of NHL3 (Fig. 1a and Extended Data Fig. 2g). We found that NHL3 interacts with SUB in this assay (Fig. 1b). To test whether SUB and NHL3 physically interact in vivo, we performed steady-state FRET-FLIM experiments in root meristem epidermal cells of 5-day-old wild-type *Arabidopsis* seedlings (Fig. 1c–g). Investigating protein interactions in vivo is of central importance in plant cell biology. FRET-FLIM is a precise and reliable method that facilitates these studies with high spatial and temporal resolution^43^. In this type of experiment, a reduction in the donor’s fluorescence lifetime indicates a physical interaction between the donor and the acceptor. As a negative control, we compared the fluorescence lifetime of p35S::LTi6a:GFP (LTi6a–GFP)^44^ in seedlings carrying LTi6a–GFP or LTi6a–GFP combined with p2x35S::LTi6b:2×mCherry (LTi6b–2×mCherry)^45^ reporters (Fig. 1d). We found that the fluorescence lifetime of LTi6a–GFP was minimally reduced in the presence of LTi6b–2×mCherry (7 picoseconds (ps)), indicating no physical interaction as expected. As a positive control, we used the induction of the physical interaction between the brassinosteroid receptor BRI1 and its co-receptor BAK1/SERK3 by the ligand brassinolide (BL)^46–51^. We assayed seedlings homozygous for pBRI1::BRI1:GFP (BRI1–GFP) or pBRI1::BRI1:GFP and pBAK1::BAK1:mCherry (BAK1–mCherry) reporters^52,53^ grown in the presence of 2μM of the brassinosteroid biosynthesis inhibitor brassinazole followed by application of 500 nM BL for 30 minutes. We observed a 2 ps fluorescence lifetime reduction for BRI1–GFP in BRI1–GFP/BAK1–mCherry seedlings exposed to brassinazole alone, whereas we determined a 65 ps lifetime reduction following application of BL (Fig. 1e), confirming previous results obtained by similar experiments^27^. This reduction in BRI1–GFP lifetime indicates physical interaction between BRI1–GFP and BAK1–mCherry. In an NHL3–SUB interaction we found a robust fluorescence lifetime reduction of 77 ps for GFP–NHL3 when combined with pUBQ::SUB:mCherry (SUB–mCherry) (Fig. 1f). The results suggest that NHL3 physically interacts with SUB in vivo.

**Extended Data Fig. 3:**
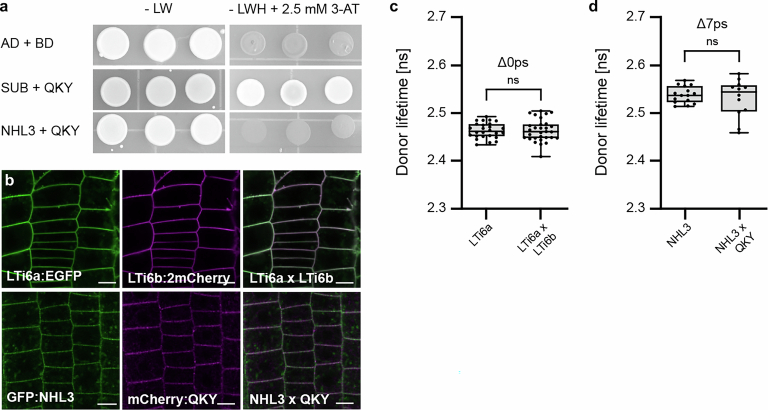
NHL3 does not interact with QKY. **a**, Yeast two-hybrid assay with the previously described (Fig. 1a) protein domains of SUB, NHL3 and QKY fused to the GAL4 activation domain (AD) and the GAL4 DNA binding domain (BD), respectively. Growth on double drop-out media (-LW) indicates successful transformation of both plasmids and on triple drop-out media (-LWH) the presence of interaction. **b**, Confocal micrographs of A. thaliana expressing the corresponding constructs and the merged image in epidermal cell files in the root meristem. **c**, Quantification of the fluorescence lifetime of LTi6a:GFP in the absence and presence of LTi6b:2mCherry at the PM. Reduction in lifetime correlates with direct physical interaction of proteins. Box-and-whisker plots show the median (horizontal line), the 25th–75th percentiles (box), and the 5th–95th percentiles (whiskers). Data are shown as mean ± s.d. for LTi6a (n = 26 individual seedlings) and LTi6a x LTi6b (n = 30 individual seedlings). Each data point represents the average of at least 5 cells per root. ns, not significant; unpaired t-test, two-tailed P-values. **d**, Quantification of the fluorescence lifetime of GFP:NHL3 in the absence and presence of mCherry:QKY at the PM. Reduction in lifetime correlates with direct physical interaction of proteins. Box-and-whisker plots show the median (horizontal line), the 25th–75th percentiles (box), and the 5th–95th percentiles (whiskers). Data are shown as mean ± s.d. for NHL3 (n = 15 individual seedlings) and NHL3 x QKY (n = 12 individual seedlings). Each data point represents the average of at least 5 cells per root. ns, not significant; unpaired t-test, two-tailed P-values. Statistical parameters and exact P values are shown in the Source data file. Similar results were obtained in at least two (**c**) or three (**d**) independent experiments. Scale bars: 8 μm. Source data

We also tested whether NHL3 interacts with QKY. However, a combination of Y2H and FRET-FLIM assays provided no evidence for a physical interaction of NHL3 with QKY (Extended Data Fig. 3).

### NHL3 controls SUB-dependent CBI responses

**Extended Data Fig. 4:**
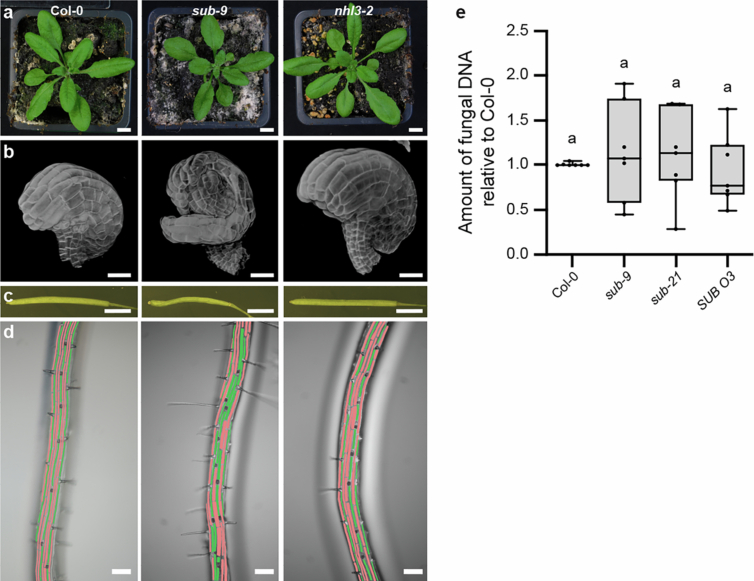
NHL3 is not involved in SUB-dependent plant development, and SUB shows no altered colonization upon *Erysiphe cruciferarum* infection. **a**–**c**, Aboveground organ morphology of Col-0, sub-9, and nhl3-2. **a**, Top view of a four-week-old rosette. Scale bars: 2 cm. **b**, Stage 3-V ovule. Scale bars: 20 μm. **c**, Stage 15 silique. Scale bars: 4 mm. **d**, Root hair cell pattern in 7-day-old seedlings. For quantification, see Extended Data Table 1. **e**, Amount of fungal DNA normalized to plant DNA upon *Erysiphe cruciferarum* infection of the indicated genotypes. Box-and-whisker plots show the median (horizontal line), the 25th–75th percentiles (box), and the 5th–95th percentiles (whiskers). Data are shown as mean ± s.d.; n = 7 biological replicates; each data point represents one independent biological replicate. Columns with no common letter are significantly different by one-way ANOVA followed by Tukey’s multiple comparison test (P < 0.05). Statistical parameters and exact P values are shown in the Source data file. Source data

We genetically tested whether *NHL3* affects *SUB*-regulated processes. *SUB* and *QKY* control root hair patterning, ovule development and silique shape^20–23^. To this end, we compared the morphological phenotypes of the *sub-21* null allele^17^ and the strong loss-of-function transfer DNA (T-DNA) insertion *nhl3-2* (refs. ^39,40^). We found no apparent morphological differences from wild type in the *nhl3-2* single mutant (Extended Data Fig. 4a–d and Extended Data Table 1). As *NHL3* is required for plant immunity^37–40^, we also examined the role of *SUB* in the seedling’s response to the powdery mildew *Erysiphe cruciferarum*. However, we failed to observe major changes in susceptibility upon *E. cruciferarum* infection (Extended Data Fig. 4e).

**Fig. 2:**
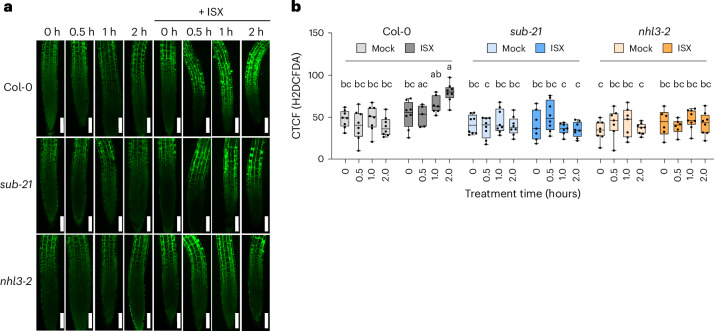
*NHL3* and *SUB* show diminished early ROS accumulation after isoxaben-induced CBI response. **a**, Confocal micrographs showing H2DCFDA signal in root tips of 6-day-old seedlings exposed to mock and 600 nM isoxaben (ISX) for the indicated time points. Genotypes are indicated. **b**, Quantification of results depicted in **a**. Box-and-whisker plots show the median (horizontal line), the 25th–75th percentiles (box) and the 5th–95th percentiles (whiskers). Data are shown as mean ± s.d. for Col-0 (*n* = 8, 8, 8, 8, 8, 5, 7 and 8 individual seedlings), *sub-21* (*n* = 8, 8, 8, 8, 7, 8, 7 and 8 individual seedlings) and *nhl3-2* (*n* = 8, 7, 7, 8, 6, 6, 9 and 8 individual seedlings) treated with 0 hour, 0.5 hour, 1 hour and 2 hours mock and isoxaben, respectively. *n* indicates the number of seedlings per treatment at the respective time point. Each data point represents one root. Columns not sharing a letter are significantly different by 2-way ANOVA followed by Tukey’s multiple comparison test (*P* < 0.05). Statistical parameters and exact *P* values are shown in the Source data. Similar results were obtained in three independent experiments. Scale bars, 100 μm. Source data

We then investigated whether *NHL3* affects the *SUB*-mediated CBI response. Isoxaben-induced phenotypes are nutrient dependent and tissue specific^10,54^. Therefore, we applied well-established isoxaben concentrations and growth conditions for plate-grown seedlings^10,11,17^. We used 7-day-old seedlings and focused on the early isoxaben-induced reactive oxygen species (ROS) accumulation and the subsequent cell wall remodelling, that is, lignin accumulation in the transition/elongation zone of the root tip and callose production in the cotyledons. All three processes are controlled by *SUB*^17^. To assess the role of *NHL3* in CBI-induced ROS production, we performed a time-course experiment in which we monitored the fluorescence intensity of the intracellular ROS probe 2′,7′-dichlorodihydrofluorescein diacetate (H2DCFDA)^55^ in roots upon treatment with mock or isoxaben (Fig. 2a,b). We confirmed previous data^17^ indicating that CBI induces an increase in ROS, starting between 30 minutes and 60 minutes. Furthermore, we found that *sub-21* and *nhl3-2* mutants failed to show this increase in ROS. These results suggest that *SUB* and *NHL3* promote early CBI-induced ROS production.

**Fig. 3:**
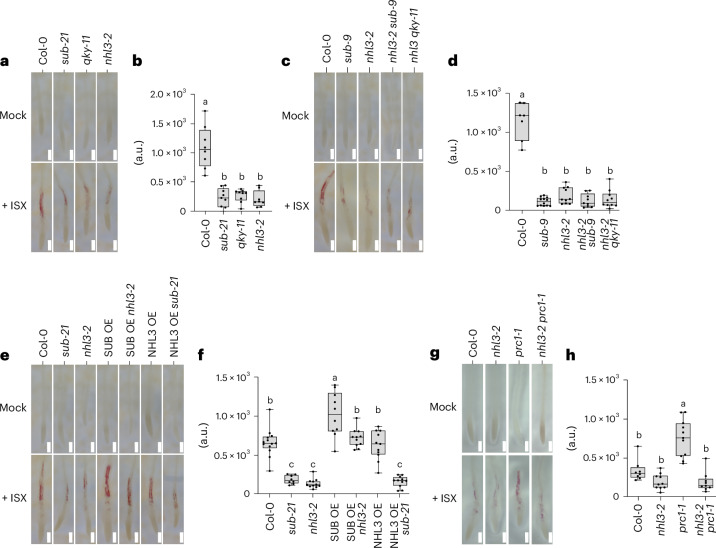
*NHL3* affects the *SUB*-dependent regulation of the isoxaben-induced CBI response. **a**,**c**,**e**,**g**, Phloroglucinol signal intensity indicating lignin accumulation in roots of 7-day-old *A. thaliana* Col-0 and mutant seedlings exposed to 600 nM isoxaben for 16 hours. Genotypes are indicated. **b**, Quantification of the results shown in **a**. Box-and-whisker plots show the median (horizontal line), the 25th–75th percentiles (box) and the 5th–95th percentiles (whiskers). Data are shown as mean ± s.d. for Col-0 and *nhl3-2* (*n* = 8 individual seedlings) and *sub-21* and *qky-11* (*n* = 9 individual seedlings), where each data point represents one root. Columns not sharing a letter are significantly different by 1-way ANOVA followed by Tukey’s multiple comparison test (*P* < 0.05). **d**, Quantification of the results shown in **c**. Data are shown as mean ± s.d. for Col-0 (*n* = 7 individual seedlings), *sub-9* (*n* = 12 individual seedlings), *nhl3-2* and *nhl3-2 qky-11* (*n* = 10 individual seedlings), and *nhl3-2* *sub-9* (*n* = 9 individual seedlings), where each data point represents one root. **f**, Quantification of the results shown in **e**. Data are shown as mean ± s.d. for Col-0 (*n* = 13 individual seedlings), *sub-21* (n = 8 individual seedlings), *nhl3-2*, *pUBQ::SUB:mCherry O3* (SUB OE) *nhl3-2*, *pUBQ::GFP:NHL3* (NHL3 OE) and NHL3 OE *sub-21* (*n* = 11 individual seedlings), and SUB OE (*n* = 10 individual seedlings), where each data point represents one root. **h**, Quantification of the results shown in **g**. Data are shown as mean ± s.d. for Col-0 (*n* = 8 individual seedlings), *nhl3-2* (*n* = 10 individual seedlings), *prc1-1* (*n* = 11 individual seedlings) and *nhl3-2 prc1-1* (*n* = 9 individual seedlings), where each data point represents 1 root. Statistical parameters and exact *P* values are shown in the Source data. Similar results were obtained from three (**b**,**d**,**f**,**h**) independent experiments. Scale bars, 100 μm. Source data

**Extended Data Fig. 5:**
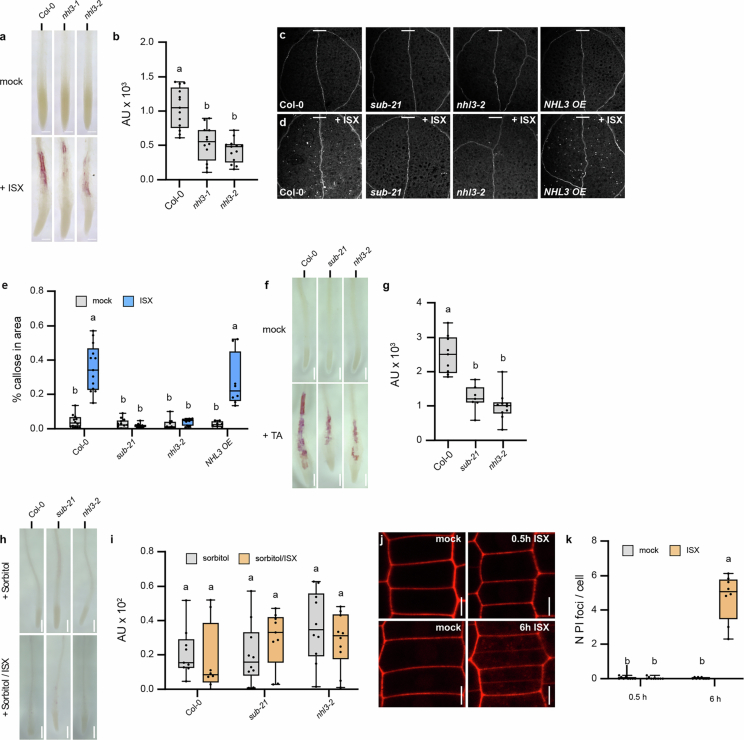
Comparison of isoxaben-induced phloroglucinol and aniline blue staining in Col-0, nhl3, and isoxaben-induced PI internalization. **a**, Phloroglucinol signal intensity indicating lignin accumulation in roots of 7 d old Col-0, nhl3-1 (SALK_035427) and nhl3-2 (SALK_150318) seedlings exposed to 600 nM isoxaben for 16 h. **b**, Quantification of the results shown in a. Box-and-whisker plots show the median (horizontal line), the 25th–75th percentiles (box), and the 5th–95th percentiles (whiskers). Data are shown as mean ± s.d. for Col-0 and nhl3-2 (n = 12 individual seedlings) and nhl3-1 (n = 12 individual seedlings). Each data point represents one root. Columns not sharing a letter are significantly different in a one-way ANOVA followed by Tukey’s multiple comparison test (P < 0.05). Scale bars: 100 μm. **c**,**d**, Confocal micrographs show cotyledons of 7 days old Col-0 and mutant seedlings treated with mock c or 600 nM isoxaben d for 24 h. The intensity of the aniline blue fluorescence signal indicates callose accumulation. **e**, Quantification of the results shown in c,d. Box-and-whisker plots show the median (horizontal line), the 25th–75th percentiles (box), and the 5th–95th percentiles (whiskers). Data are shown as mean ± s.d. for Col-0 (n = 12/13 cotyledons), sub-21 (n = 13/12 cotyledons), nhl3-2 (n = 12/13) and NHL3 OE (n = 8/8) treated with mock and isoxaben, respectively. Each data point represents one cotyledon. Columns not sharing a letter are significantly different in a two-way ANOVA followed by Tukey’s multiple comparison test (P < 0.05). Scale bars: 200 μm. **f**, Phloroglucinol signal intensity indicating lignin accumulation in roots of 7 days old Col-0 and mutant seedlings exposed to 200 nM thaxtomin A for 24 h. Genotypes are indicated. **g**, Quantification of the results shown in f. Box-and-whisker plots show the median (horizontal line), the 25th–75th percentiles (box), and the 5th–95th percentiles (whiskers). Data are shown as mean ± s.d. for Col-0 (n = 9 individual seedlings), sub-21 (n = 7 individual seedlings) and nhl3-2 (n = 10 individual seedlings). Each data point represents one root. Columns not sharing a letter are significantly different by one-way ANOVA followed by Tukey’s multiple comparison test (P < 0.05). **h**, Phloroglucinol signal intensity indicating lignin accumulation in roots of 7 d old A. thaliana Col-0 and mutant seedlings exposed to 300 mM Sorbitol or 300 mM Sorbitol with 600 nM isoxaben for 16 h. Genotypes are indicated. **i**, Quantification of the results shown in h. Box-and-whisker plots show the median (horizontal line), the 25th–75th percentiles (box), and the 5th–95th percentiles (whiskers). Data are shown as mean ± s.d. for Col-0 (n = 9/8 individual seedlings), sub-21 (n = 10/9 individual seedlings) and nhl3-2 (n = 10/10 individual seedlings) treated with only sorbitol and both sorbitol and isoxaben, respectively. Each data point represents one root. Columns not sharing a letter are significantly different by two-way ANOVA followed by Tukey’s multiple comparison test (P < 0.05). Scale bars: 200 μm. **j**, Fluorescence micrographs show optical sections of epidermal cells of the root meristem of 5 d old seedlings stained with PI after mock and isoxaben treatment. Note increased PI signal after 6 h treatment with isoxaben. **k**, Quantification of the results shown in j. Box-and-whisker plots show the median (horizontal line), the 25th–75th percentiles (box), and the 5th–95th percentiles (whiskers). Data are shown as mean ± s.d. for mock (n = 9/10 individual seedlings) and isoxaben (n = 9/8 individual seedlings) treated for 0.5 h and 6 h, respectively. Each data point represents one root. Columns not sharing a letter are significantly different by two-way ANOVA followed by Tukey’s multiple comparison test (P < 0.05). Brightness was uniformly adjusted across all images using Fiji. Statistical parameters and exact P values are shown in the Source data file. Similar results were obtained from at least two (**i**,**k**) or three (**b**,**e**,**g**) independent experiments. Scale bars: 8 μm. Source data

The occurrence of lignin or callose was inferred from phloroglucinol and aniline blue staining, respectively. Lignification begins around 4–6 hours after the onset of CBI but is robustly detected after about 12–16 hours of isoxaben exposure^10^. We observed that a 16-hour exposure to 600 nM isoxaben induced a readily detectable phloroglucinol signal in wild type, whereas the *nhl3-2* mutant showed a marked decrease in signal comparable to the reduced signal in the strong loss-of-function alleles *sub-9*, *sub-21* or *qky-11* (refs. ^22,24^) (Fig. 3a–d). A second independent T-DNA insertion loss-of-function allele of *NHL3*, *nhl3-1* (refs. ^40,56^), showed equivalent signal reduction (Extended Data Fig. 5a,b). No further signal decrease was detected in *nhl3-2 sub-9* or *nhl3-2 qky-11* double mutants. A comparable reduction in isoxaben-induced callose accumulation in cotyledons was also observed in *sub-21* and *nhl3-2* mutants (Extended Data Fig. 5c–e).

Overexpression of SUB results in hyperaccumulation of the isoxaben-induced phloroglucinol signal^17^ (Fig. 3e,f). To test whether this effect depends on *NHL3*, we crossed the line pUBQ::SUB:mCherry O3 (*OE::SUB O3*)^17^ with *nhl3-2* and analysed the phenotype of a line homozygous for the *nhl3-2* mutation and homozygous for the pUBQ::SUB:mCherry O3 construct (*OE::SUB O3 nhl3-2*). We found the signal strength comparable to the wild type. This indicates that the hyperaccumulation of lignin upon overexpression of *SUB* requires *NHL3*. It further shows that overexpression of *SUB* rescues the *nhl3-2* mutant phenotype. Next, we examined the effect of *NHL3* overexpression. We observed that lines transgenic for the pUBQ::GFP:NHL3 reporter (*OE::NHL3*) showed a phloroglucinol signal equivalent to wild type (Fig. 3f). We crossed the *OE::NHL3* line C1 with *sub-21* and analysed the phenotype of *OE::NHL3 sub-21* seedlings. We found the signal strength was comparable to *sub-21*, indicating that the signal in *OE::NHL3* C1 depends on *SUB*.

To exclude an isoxaben-specific effect, we treated seedlings with thaxtomin A, another cellulose biosynthesis inhibitor^57,58^. Treatment of wild-type seedlings with 200 nM thaxtomin A for 24 hours resulted in prominent phloroglucinol signal in root tips (Extended Data Fig. 5f,g). However, similar treatment of *sub-21* and *nhl3-2* seedlings resulted in a strongly diminished signal. This result indicates that *SUB* and *NHL3* are required for root tip lignification induced by thaxtomin A.

Next, we examined the impact of genetically reducing cellulose synthesis. Cellulose is synthesized by cellulose synthases, protein complexes composed of diverse and partially redundant CESA subunits^7,59^. The CESA6 subunit is encoded by *PROCUSTE 1* (*PRC1*), and *prc1-1* mutants show reduced cellulose levels^60^. In our assay, untreated light-grown *prc1-1* seedlings failed to show root tip lignification, as evidenced by an absence of detectable phloroglucinol staining, which corroborates previous findings^14^. Under these conditions, the reduction in cellulose levels in *prc1-1* is insufficient to induce root tip lignification, which is probably due to the presence of compensating CESA complexes. Applying 50 nM isoxaben to wild-type or *nhl3-2* seedlings resulted in a minimal phloroglucinol signal in the root tips, suggesting that this concentration of isoxaben only marginally attenuates cellulose synthase activity (Fig. 3g,h). In contrast, exposing *prc1-1* seedlings to 50 nM isoxaben resulted in a strong phloroglucinol signal, indicating prominent lignification. However, treating *prc1-1 nhl3-2* double mutants did not result in any difference compared with wild type or *nhl3-2* single mutants. These results suggest that *NHL3* plays a role in root tip lignification in response to reduced cellulose levels caused by the absence of *PRC1*/*CESA6* activity.

The presence of osmolytes, such as sorbitol, inhibits the build-up of lignin and callose, which are normally triggered by isoxaben. This reveals that the isoxaben-induced cell wall damage response depends on turgor pressure^10,11,61^. The *SUB*-mediated CBI response is sensitive to sorbitol^17^. Thus, we examined the phloroglucinol signal in wild type and seedlings lacking *SUB* or *NHL3* activity exposed to 600 nM isoxaben and 300 mM sorbitol. We observed that simultaneous application of isoxaben and sorbitol eliminated the phloroglucinol signal in all instances (Extended Data Fig. 5h,i). These results indicate that *SUB* and *NHL3* do not affect osmoperception in this assay.

In conclusion, our combined pharmacological and genetic data suggest that *NHL3* promotes cell wall remodelling after CBI-induced cell wall damage. Furthermore, they indicate that *NHL3* acts in the same genetic pathway as *SUB* and *QKY* regarding CBI-induced lignin accumulation in seedling root tips, and as *SUB* with respect to callose formation in cotyledons. However, *NHL3* does not take part in developmental processes, such as root hair patterning or ovule development, which rely on *SUB* and *QKY* function.

### Continuous isoxaben application modifies pectin

The cell wall is a complex structure with intricate interactions between its various constituents^6,62^. For example, reduced cellulose biosynthesis can lead to pectin defects in the seed coat^63^. We examined whether isoxaben treatment alters the pectin component of the cell wall in epidermal cells of the seedling root meristem. Propidium iodide (PI) labels demethylesterified pectin in *Arabidopsis* pollen tubes and root hairs^64^. We investigated whether isoxaben treatment would induce cytoplasmic PI-labelled puncta, which would indicate internalized vesicles containing pectin fragments. After 30 minutes of isoxaben treatment, we did not observe such puncta. However, after 6 hours, we noticed a significant presence of cytoplasmic PI-labelled puncta (Extended Data Fig. 5j,k). These data suggest that a 6-hour isoxaben treatment induces pectin endocytosis and changes the status of cell wall pectin.

### NHL3 does not affect SUB oligomerization

**Fig. 4:**
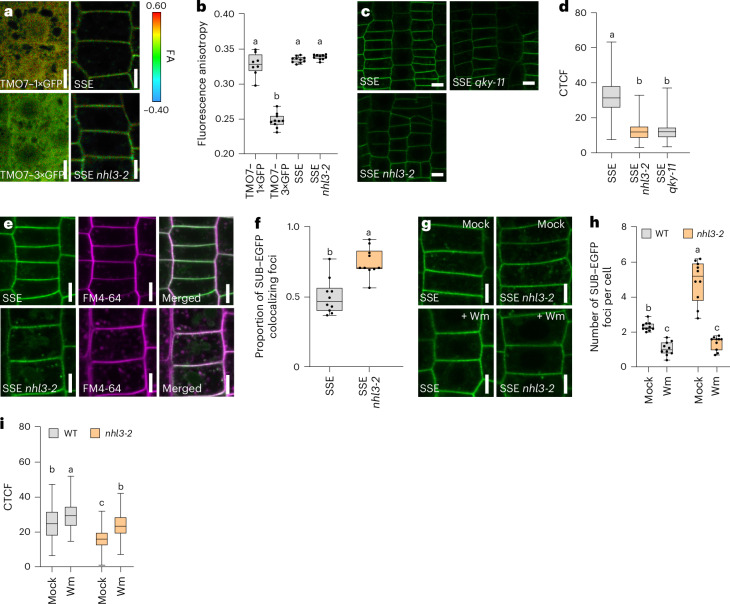
NHL3 does not affect SUB oligomerization and maintains SUB levels at the plasma membrane. **a**,**b**, FA analysis in root epidermal cells of 5-day-old seedlings. **a**, Images show TMO7 fused to two different GFP moieties (TMO7–1×GFP and TMO7–3×GFP) and pSUB::SUB:EGFP (SSE) reporters. Note the reduced anisotropy in TMO7–3×GFP. Genotypes are indicated. **b**, Quantification of the data shown in **a**. Box-and-whisker plots show the median (horizontal line), the 25th–75th percentiles (box) and the 5th–95th percentiles (whiskers). Data are shown as mean ± s.d. for TMO7–1×GFP (*n* = 8 individual seedlings) and TMO7–3×GFP, SSE and SSE *nhl3-2* (*n* = 10 individual seedlings), where each data point represents the average of at least 6 cells per root. Columns not sharing a letter are significantly different in a 1-way ANOVA followed by Tukey’s multiple comparison test (*P* < 0.05). Similar results were obtained in at least three independent experiments. **c**, Signal intensity of a functional SSE reporter at the plasma membrane in root epidermal cells of 5-day-old seedlings in corresponding mutant backgrounds. Genotypes are indicated. **d**, Quantification of data from **c**. Box-and-whisker plots show the corrected total cell fluorescence (CTCF) at the plasma membrane with the median (horizontal line), the 25th–75th percentiles (box) and the 5th–95th percentiles (whiskers). Data are shown as mean ± s.d. for SSE (*n* = 100 cells), SSE *nhl3-2* (*n* = 150 cells) and SSE *qky-11* (*n* = 120 cells), where each data point represents the plasma membrane of one cell in 10–15 individual seedlings. Columns not sharing a letter are significantly different in a 1-way ANOVA followed by Tukey’s multiple comparison test (*P* < 0.05). **e**, Fluorescence micrographs show optical sections of epidermal cells of the root meristem of 5-day-old seedlings with partial colocalization of SSE and FM4-64 foci after treatment with FM4-64 for 5 minutes. Genotypes are indicated. **f**, Quantification of data from **e**. Data are shown as mean ± s.d. with *n* = 10 individual seedlings, where each data point represents the average of at least 6 cells per root. Columns not sharing a letter are significantly different in an unpaired two-tailed *t* test (*P* < 0.05). **g**, Fluorescence micrographs showing the subcellular localization of the SSE signal from 5-day-old seedlings in the presence of wortmannin (Wm) and DMSO (mock). Genotypes are indicated. **h**, Quantification of the data in **g**. Box-and-whisker plots showing the number of SSE endosomes per cell with the median (horizontal line), the 25th–75th percentiles (box) and the 5th–95th percentiles (whiskers). Data are shown as mean ± s.d. with *n* = 10 individual seedlings, where each data point represents the average of at least 6 cells per root. Columns with no common letter are significantly different by two-way ANOVA followed by Šidák’s multiple comparison test (*P* < 0.05). **i**, Quantification of the data in **g**. Box-and-whisker plots showing CTCF at the plasma membrane with the median (horizontal line), the 25th–75th percentiles (box) and the 5th–95th percentiles (whiskers). Data are shown as mean ± s.d. with wild type (WT; *n* = 99 cells) and *nhl3-2* (*n* = 100 cells), where each data point represents the plasma membrane of 1 cell in 10 different roots. Columns with no common letter are significantly different by two-way ANOVA followed by Šidák’s multiple comparison test (*P* < 0.05). Statistical parameters and exact *P* values are shown in the Source data. Similar results were obtained from at least two (**f**) or three (**b**,**d**,**h**,**i**) independent experiments. Scale bars, 8 μm. Source data

Previous data suggested that SUB forms homomultimers in a QKY-dependent fashion and that isoxaben treatment attenuates SUB oligomerization^13,27^. We tested if NHL3 affects SUB oligomerization by comparing the results of steady-state fluorescence anisotropy (FA) measurements of SUB–EGFP in root meristem epidermal cells of 5-day-old wild-type and *nhl3-2* seedlings. FA provides an estimate of the rotational freedom of a fluorescence reporter, such as SUB–EGFP. In the case of homo-oligomerization, homo-FRET can occur between the GFPs, resulting in a reduction of the FA value compared with the monomers^43,65^. Control experiments used lines expressing the TARGET OF MP7 (TMO7) transcription factor fused to either one (TMO7–1×GFP) or three (TMO7–3×GFP) GFP moieties^66^. TMO7–1×GFP displays restricted rotational dynamics, whereas TMO7–3×GFP shows reduced anisotropy attributable to homo-FRET between adjacent GFP moieties (Fig. 4a,b). We observed an FA value for SUB–EGFP of 0.334 in wild type, confirming previous results^13^, and of 0.338 in *nhl3-2* (Fig. 4a,b). This minimal difference in FA values indicates that NHL3, unlike QKY, does not promote SUB homomultimerization.

### NHL3 maintains SUB at the plasma membrane

**Extended Data Fig. 6:**
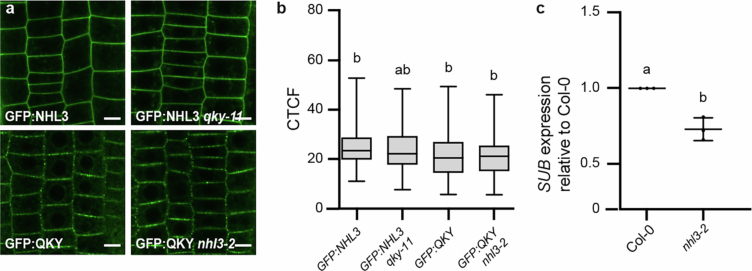
The presence of GFP:NHL3 and GFP:QKY at the plasma membrane is independent of each other and NHL3 affects SUB expression. **a**, Signal intensity of functional GFP:NHL3 and GFP:QKY reporters at the PM in root epidermal cells of 5 d old seedlings in corresponding mutant backgrounds. Genotypes are indicated. Scale bars: 8 μm. **b**, Quantification of data from a. Box-and-whisker plots showing corrected total cell fluorescence (CTCF) at the plasma membrane with the median (horizontal line), the 25th–75th percentiles (box), and the 5th–95th percentiles (whiskers). Data are shown as mean ± s.d. for NHL3 (n = 98 cells), NHL3 qky-11 (n = 99 cells), QKY and QKY nhl3-2 (n = 80 cells). Plasma membrane signals from 8–10 individual cells per root were analyzed for a total of 8–10 roots. Columns not sharing a letter are significantly different by one-way ANOVA followed by Tukey’s multiple comparison test (P < 0.05). The experiment was performed three times with essentially identical results. **c**, RT-qPCR of SUB in Col-0 and nhl3-2 in 7 d old seedlings. Mean ± SD is shown, n = 3 biological replicates. Data points designate results of three individual biological replicates. Statistical parameters and exact P values are shown in the Source data file. Source data

SUB was reported to undergo constitutive internalization^31,33^. We asked whether NHL3 is involved in the regulation of SUB internalization in epidermal cells of the root meristem of 5-day-old seedlings. As a control, we tested whether QKY and NHL3 mutually influence each other’s presence on the cell surface. To this end, we crossed pUBQ::GFP:QKY (GFP–QKY) and GFP–NHL3 reporter lines with *nhl3-2* and *qky-11*, respectively, and compared the signal strength of the reporters in the mutants with that in the wild type. Compared with wild type, we found similar signal levels of GFP–QKY in *nhl3-2* and GFP–NHL3 in *qky-11* (Extended Data Fig. 6a,b). These results indicate that the presence of NHL3 at the cell surface is not dependent on QKY and vice versa.

We then crossed the SUB–EGFP Col-0 line with *qky-11* and *nhl3-2*, selected the homozygous mutants carrying two copies of the transgene and compared the intensity of the SUB–EGFP reporter signal at the plasma membrane between wild type and mutants (Fig. 4c,d). We found a similar reduction in reporter signal in *nhl3-2* and *qky-11*. To assess whether the decrease in plasma membrane signal of the SUB–EGFP reporter in *nhl3-2* was associated with increased internalization of SUB–EGFP, we imaged cells after a 5-minute treatment with the endocytic tracer dye FM4-64 and scored the cytoplasmic puncta showing both SUB–EGFP and FM4-64 signal (Fig. 4e,f). Colocalization was defined using a previously established criterion (distance between the 2 centres of the signals below the resolution limit of the objective, that is, less than 0.24 μm)^31,67^. We found that 52.3% (*n* = 232) of all cytoplasmic SUB–EGFP puncta were also labelled with FM4-64 in wild type, while 67.7% (*n* = 401) of puncta showed SUB–EGFP/FM4-64 colabelling in *nhl3-2*. Treatment of seedlings with 33 μM of the phosphatidylinositol 3-kinase inhibitor wortmannin, which interferes with vesicle formation at the plasma membrane^67–69^, for 60 minutes caused a decrease in internal SUB–EGFP puncta and an increase in reporter signal at the plasma membrane in wild type and *nhl3-2* (Fig. 4g–i).

*QKY* does not regulate *SUB* transcript levels^33^. To assess the role of *NHL3* in this process, we examined whether *NHL3* affects *SUB* expression. We found that *SUB* transcript levels were slightly reduced in *nhl3-2* seedlings (Extended Data Fig. 6c). However, this minor reduction is insufficient to result in the root hair patterning defects observed in *sub* homozygous or heterozygous mutants^13^. The combined results suggest that *NHL3* is required for maintaining SUB at the plasma membrane and supporting wild-type *SUB* expression.

**Extended Data Fig. 7:**
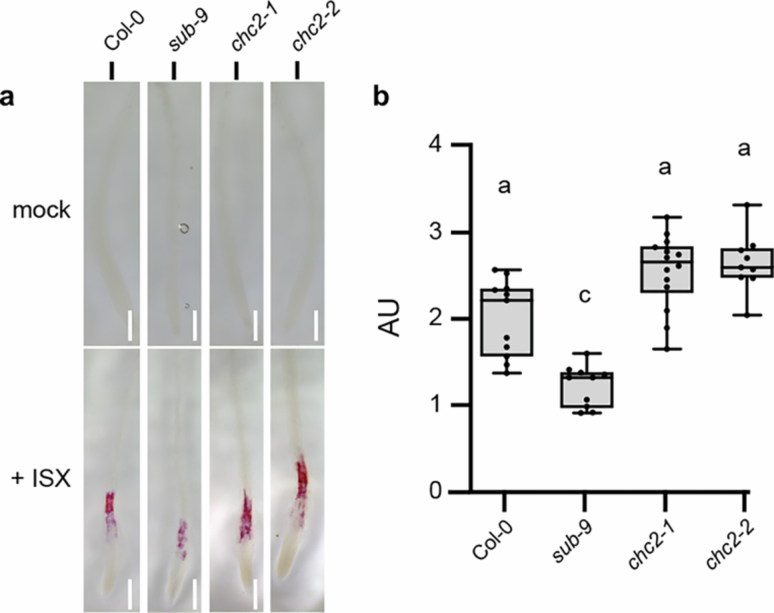
Plants with reduced CHC2 activity show hyper lignification during isoxaben-induced CBI response. **a**, Phloroglucinol signal intensity indicating lignin accumulation in roots of 7 d old A.thaliana Col-0 and mutant seedlings exposed to 600 nM isoxaben for 16 h. Genotypes are indicated. **b**, Quantification of the results shown in a. Box-and-whisker plots show the median (horizontal line), the 25th–75th percentiles (box), and the 5th–95th percentiles (whiskers). Data are shown as mean ± s.d. for Col-0 (n = 11 individual seedlings), sub-9 (n = 10 individual seedlings), chc2-1 (n = 14 individual seedlings) and chc2-2 (n = 9 individual seedlings). Each data point represents one root. Columns not sharing a letter are significantly different by one-way ANOVA followed by Tukey’s multiple comparison test (P < 0.05). The experiment was performed two times with similar results. Statistical parameters and exact P values are shown in the Source data file. Scale bars: 100 μm. Source data

Next, we addressed the question of whether SUB functions at the plasma membrane. SUB internalization was previously shown to be clathrin dependent^31^. Budding cargo-containing clathrin-coated vesicles are enclosed within a lattice of clathrin triskelia formed by clathrin heavy chains (CHCs) and clathrin light chains^70^. In *Arabidopsis*, CHCs are encoded by two probably redundant genes, *CLATHRIN HEAVY CHAIN 1* (*CHC1*) and *CHC2* ^71^. Both SUB and CHCs were present in the same complex in vivo, and mutations in *CHC2* resulted in decreased SUB internalization and root hair patterning defects comparable to *SUB* overexpression^31,72^. We tested whether *CHC2* is required for the CBI response. To this end, we investigated isoxaben-induced lignin accumulation in the *chc2-1* and *chc2-2* mutants^73^. We found an elevated phloroglucinol signal in both mutants compared with the wild type, indicating that *CHC2* is necessary for the correct CBI response. The effects are similar to those observed with *SUB* overexpression (Fig. 3e and Extended Data Fig. 7a,b). These data support the idea that SUB functions at the plasma membrane rather than at endosomes, for example.

### NHL3 and QKY maintain SUB at the plasma membrane

**Fig. 5:**
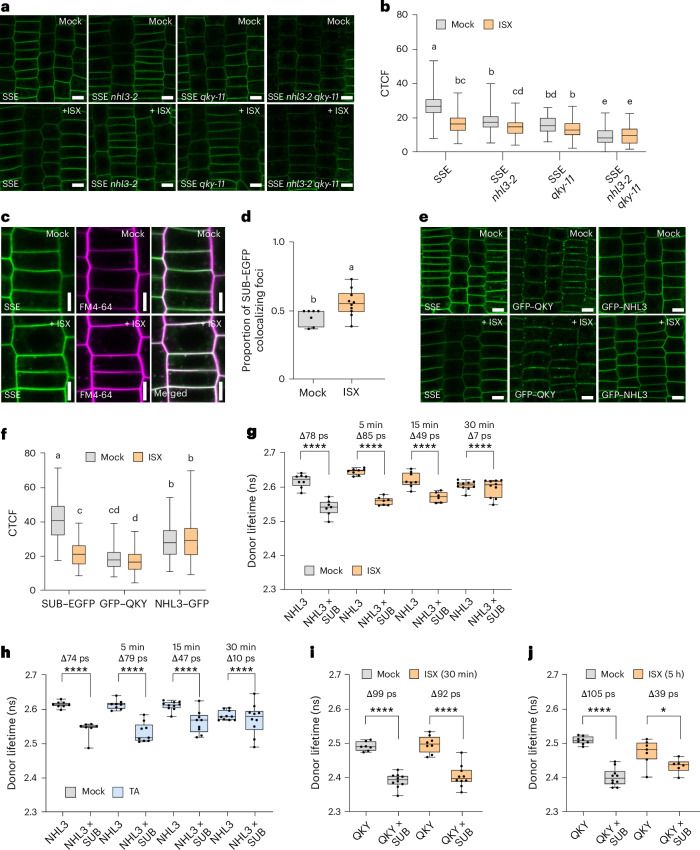
CBI disrupts the physical interaction of SUB with NHL3 and QKY. **a**, Signal intensity of a functional pSUB::SUB:EGFP reporter (SSE) at the plasma membrane in root epidermal cells of 5-day-old seedlings after exposure to mock and isoxaben for 5 hours. Genotypes are indicated. **b**, Quantification of data from **a**. Box-and-whisker plots showing CTCF at the plasma membrane with the median (horizontal line), the 25th–75th percentiles (box) and the 5th–95th percentiles (whiskers). Data are shown as mean ± s.d. for SSE (*n* = 88 and 90 cells), SSE *nhl3-2* (*n* = 99 and 86 cells), SSE *qky-11* (*n* = 100 and 95 cells) and SSE *nhl3-2 qky-11* (*n* = 50 and 50 cells) treated with mock and isoxaben, respectively. Each data point represents the plasma membrane of one cell in five to ten different roots. Columns with no common letter are significantly different by 2-way ANOVA followed by Tukey’s multiple comparison test (*P* < 0.05). **c**, Fluorescence micrographs show optical sections of epidermal cells of the root meristem of 5-day-old seedlings with partial colocalization of SSE and FM4-64 foci after treatment with FM4-64 for 5 minutes. **d**, Quantification of the data in **c**. Box-and-whisker plots show the median (horizontal line), the 25th–75th percentiles (box) and the 5th–95th percentiles (whiskers). Data are shown as mean ± s.d. with mock (*n* = 7 individual seedlings) and isoxaben (*n* = 10 individual seedlings), where each data point represents the average of at least 6 cells per root. Columns not sharing a letter are significantly different in an unpaired 2-tailed *t* test (*P* < 0.05). **e**, Signal intensity of SSE, GFP–QKY (pUBQ::GFP:QKY) and GFP–NHL3 (pUBQ::GFP:NHL3) reporters at the plasma membrane in root epidermal cells of 5-day-old seedlings after exposure to mock and isoxaben for 5 hours. Genotypes are indicated. **f**, Quantification of data in **e**. Box-and-whisker plots showing CTCF at the plasma membrane with the median (horizontal line), the 25th–75th percentiles (box) and the 5th–95th percentiles (whiskers). Data are shown as mean ± s.d. for SUB–EGFP (*n* = 98 and 99 cells), GFP–QKY (*n* = 77 and 99 cells) and GFP–NHL3 (*n* = 100 and 110 cells) treated with mock and isoxaben, respectively. Each data point represents the plasma membrane of one cell in five to ten different roots. Columns with no common letter are significantly different by 2-way ANOVA followed by Šidák’s multiple comparison test (*P* < 0.05). **g**, Quantification of the fluorescence lifetime of GFP–NHL3 in the absence and presence of SUB (pUBQ::SUB:mCherry) at the plasma membrane after treatment with mock or isoxaben for 5 minutes, 15 minutes and 30 minutes. Reduction in lifetime indicates physical interaction of proteins. Box-and-whisker plots show the median (horizontal line), the 25th–75th percentiles (box) and the 5th–95th percentiles (whiskers). Data are shown as mean ± s.d. with NHL3 (*n* = 8, 8, 8 and 10 individual seedlings) and NHL3 × SUB (*n* = 7, 7, 6 and 10 individual seedlings) treated with mock and isoxaben (5 minutes, 15 minutes and 30 minutes), respectively. *n* indicates number of seedlings per treatment and consecutive timepoint. Each data point represents the average of at least five cells per root. One-way ANOVA followed by post hoc Šidák’s multiple comparison test. **h**, Quantification of the fluorescence lifetime of GFP–NHL3 in the absence and presence of SUB (pUBQ::SUB:mCherry) at the plasma membrane after treatment with mock or thaxtomin A for 5 minutes, 15 minutes and 30 minutes. Reduction in lifetime indicates physical interaction of proteins. Data are shown as mean ± s.d. with NHL3 (*n* = 8, 8, 10 and 10 individual seedlings) and NHL3 × SUB (*n* = 7, 9, 9 and 10 individual seedlings) treated with mock and isoxaben (5 minutes, 15 minutes and 30 minutes), respectively. Each data point represents the average of at least five cells per root. One-way ANOVA followed by post hoc Šidák’s multiple comparison test. **i**, Quantification of the fluorescence lifetime of QKY (pUBQ::GFP:QKY) in the absence and presence of SUB (pUBQ::SUB:mCherry) at the plasma membrane after treatment with mock or isoxaben for 30 minutes. Reduction in lifetime indicates physical interaction of proteins. Data are shown as mean ± s.d. with QKY (*n* = 7 and 8 individual seedlings) and QKY × SUB (*n* = 10 and 10 individual seedlings) treated with mock and isoxaben for 30 minutes, respectively. Each data point represents the average of at least five cells per root. One-way ANOVA followed by post hoc Tukey’s multiple comparison test. **j**, Quantification of the fluorescence lifetime of QKY (pUBQ::GFP:QKY) in the absence and presence of SUB (pUBQ::SUB:mCherry) at the plasma membrane after treatment with mock or isoxaben for 5 hours. Reduction in lifetime indicates physical interaction of proteins. Data are shown as mean ± s.d. with QKY (*n* = 8 and 7 individual seedlings) and QKY × SUB (*n* = 10 and 6 individual seedlings) treated with mock and isoxaben for 5 hours, respectively. Each data point represents the average of at least five cells per root. One-way ANOVA followed by post hoc Šidák’s multiple comparison test. Similar results were obtained from at least two (**d**) or three (**b**,**f**–**j**) independent experiments. Scale bars, 8 μm. *P < 0.05, ***P < 0.001, ****P < 0.0001. Source data

The available data suggest that both QKY and NHL3 promote SUB’s presence at the plasma membrane^13,33^ (Fig. 4c,d). We next investigated the relative contributions of NHL3 and QKY in this process. To assess whether QKY and NHL3 function in the same pathway, we crossed SUB–EGFP into *qky-11*, *nhl3-2* and *qky-11 nhl3-2* and monitored the plasma membrane signal of SUB–EGFP in epidermal cells of the root meristem of mock-treated 5-day-old wild-type and mutant seedlings. Compared with wild type, we found reduced reporter signals in *qky-11* and *nhl3-2*, while *qky-11 nhl3-2* double mutants showed an even more reduced signal (Fig. 5a,b). This additive effect suggests that QKY and NHL3 function in separate pathways and that both are required for plasma membrane localization of SUB.

### CBI induces rapid internalization of SUB

Treatment with isoxaben results in greater internalization of SUB than under stress-free conditions and partial elimination of SUB from the plasma membrane^13^. In 5-day-old seedlings, isoxaben-induced SUB internalization is clearly visible after about 5 hours of treatment with 600 nM isoxaben^13^ (Fig. 5a,b). We tested whether the induction of SUB internalization by isoxaben starts at an earlier time point. We examined the number of internalized SUB–EGFP puncta in the epidermal cells of the root meristem of 5-day-old seedlings after treatment with 600 nM isoxaben for 30 minutes and adding 1 μM FM4-64 for the final 5 minutes. We found a slight but noticeable increase in internalized SUB–EGFP puncta (58.7%, *n* = 269) compared with the mock-treated wild type (44.3%, *n* = 165), indicating that CBI-induced internalization of SUB is a rapid response that starts much earlier than previously thought (Fig. 5c,d).

### CBI internalizes SUB by disrupting the NHL3–QKY complex

How CBI mediates SUB internalization is not understood. Therefore, we investigated whether and, if so, how CBI impairs the role of NHL3 and QKY in the maintenance of SUB at the plasma membrane. We first examined whether isoxaben treatment resulted in a reduced cell surface signal of GFP–QKY or GFP–NHL3 and found no effect under our assay conditions (Fig. 5e,f). We then monitored the SUB–EGFP reporter signal in epidermal cells of the root meristem of 5-day-old seedlings of wild type, *qky-11*, *nhl3-2* and *qky-11 nhl3-2* treated with 600 nM isoxaben for 5 hours. We found that the reporter signal in isoxaben-treated wild type was similar to its intensity observed in mock-treated *qky-11* and *nhl3-2* mutants. Isoxaben treatment only slightly, if at all, further reduced the cell surface signal of SUB–EGFP in *qky-11* or *nhl3-2* (Fig. 5a,b). Furthermore, treatment with isoxaben did not reduce the plasma membrane reporter signal below the already greatly reduced level in the mock-treated *qky-11 nhl3-2* double mutants (Fig. 5a,b). Thus, isoxaben has no effect on SUB–EGFP localization in the double-mutant background.

Next, we examined whether CBI affects the physical interaction of SUB with NHL3 and/or QKY. To this end, we performed steady-state FRET-FLIM experiments in root meristem epidermal cells of 5-day-old seedlings carrying the GFP–NHL3/SUB–mCherry reporter configuration after 5 minutes, 15 minutes and 30 minutes of exposure to 600 nM isoxaben or 200 nM thaxtomin A. We observed that the reduction in donor lifetime became noticeably smaller around 15 minutes after the start of isoxaben or thaxtomin A treatment. By 30 minutes of treatment, the reduction was undetectable (Fig. 5g,h). In contrast, a prominent reduction in donor lifetime was still observable for the GFP–QKY/SUB–mCherry pair after 30 minutes of exposure to isoxaben compared with mock (Fig. 5i,j). However, after 5 hours of isoxaben application, we noticed that the lifetime of the GFP–QKY donor was substantially reduced to less than half that of the mock value. The results suggest that exposure to isoxaben or thaxtomin A rapidly dissociates the SUB–NHL3 complex. In contrast, isoxaben causes a delayed and less drastic reduction in the SUB–QKY interaction.

## Discussion

NHL3 was previously shown to participate in pattern-triggered plant immunity^37–40^ and cell wall remodelling induced by the perception of pathogens^40^. Our results identify NHL3 as a new factor involved in the CBI-induced cell wall damage response.

Furthermore, our data provide molecular insight into how NHL3 controls cell wall remodelling during the CBI response. They indicate that NHL3 is required for the SUB-dependent CBI response. NHL3 directly interacts with SUB via their intracellular domains, maintaining SUB at the plasma membrane by counteracting its constitutive clathrin-dependent internalization. This ensures sufficient signalling capacity to respond immediately to cell wall damage. Supporting this, SUB overexpression rescues the *nhl3-2* phenotype by compensating for reduced cell surface levels. Isoxaben or thaxtomin A disrupts the SUB–NHL3 interaction within 15 minutes, triggering increased SUB internalization. NHL3 is necessary for the CBI-induced and SUB-mediated accumulation of ROS within 30–60 minutes of treatment. As isoxaben-induced CESA loss occurs within 20 minutes (ref. ^74^), this process is part of the early CBI response. In addition, NHL3 promotes later SUB-dependent and CBI-induced cell wall remodelling including lignin and callose accumulation. In summary, we propose that NHL3 stabilizes SUB at the plasma membrane. CBI triggers rapid SUB activation, followed by the disruption of the SUB–NHL3 complex. This induces SUB endocytosis through ligand-induced receptor-mediated internalization, which ultimately attenuates the signalling response.

NHL3 and QKY bind directly to SUB in *Arabidopsis* and, similarly to NHL3, QKY is involved in maintaining SUB at the plasma membrane^13,33^. How do they relate to each other? Our genetic evidence suggests that *NHL3* and *QKY* do not have overlapping functions under non-stress conditions. Moreover, *NHL3* positively controls the transcript levels of *SUB*, whereas *QKY* is not required for this process^33^. In addition, QKY, but not NHL3, promotes SUB homo-oligomerization. There are also marked differences between *NHL3* and *QKY* function in the CBI response. Both are required for root tip lignification; however, *NHL3* is also required for callose production in the cotyledons, whereas *QKY* is not.

The response to CBI-induced cell wall damage involves multiple hierarchical steps^15,19^. How CBI triggers SUB internalization and blocks SUB homo-oligomerization was not understood^13^. We show here that CBI not only rapidly disrupts the physical interaction of SUB with NHL3 within 15 minutes but also eventually alters its interaction with QKY after approximately 5 hours. This change in the architecture of the SUB–QKY complex can explain the concurrent increase in SUB internalization. Furthermore, as SUB homo-oligomerization depends on QKY^27^, it could also account for the CBI-induced inhibition of this process.

What could be the basis for the differences in responses? A reduction in cellulose biosynthesis is known to affect the pectin component of the cell wall^60,63^. In addition, our data suggest that 6 hours of CBI results in the internalization of pectin fragments. It is thus conceivable that a reduction of cellulose biosynthesis secondarily leads to further changes in the biochemistry and architecture of the cell wall. We hypothesize that CBI-induced changes in the SUB–QKY complex architecture are due to signalling responses to such secondary alterations in the cell wall. In accordance with this notion, exposing seedlings to epigallocatechin gallate, an inhibitor of pectin methylesterase activity^75^, reduced *SUB* transcript and SUB protein levels and caused defects in root hair patterning^13^.

**Extended Data Fig. 8:**
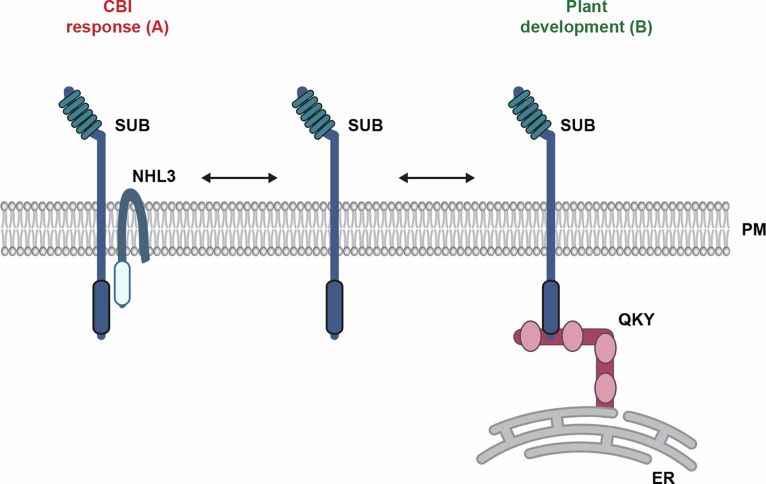
Cartoon depicting the proposed multiple, structurally and functionally distinct SUB-complexes. Abbreviations: ER, endoplasmic reticulum; PM, plasma membrane.

With its small extracellular domain of six leucine-rich repeats^76^, SUB resembles co-receptors like BRI1-ASSOCIATED RECEPTOR KINASE 1 (BAK1; also known as SOMATIC EMBRYOGENESIS RECEPTOR-LIKE KINASE 3 (SERK3)), which modulate development, hormone regulation and stress responses^77^. We propose SUB acts as a multifunctional co-receptor for both development and cellulose biosynthesis inhibitor-induced cell wall remodelling via at least two distinct surface complexes (Extended Data Fig. 8). Complex A (SUB–NHL3) primarily drives the CBI response, including ROS production, callose accumulation and lignification. Complex B (SUB–QKY) mainly controls developmental pathways, such as root hair patterning and ovule morphogenesis, with a minor role in CBI-induced lignification. SUB is maintained at the plasma membrane through direct binding to NHL3 and QKY, which prevents its internalization. CBI modulates SUB activity by triggering receptor-mediated endocytosis through two distinct processes. An early rapid process inhibits the SUB–NHL3 interaction, causing quick internalization of complex A, while a later-acting process attenuates the SUB–QKY interaction, resulting in internalization of complex B and blocking of SUB oligomerization. This CBI-induced disruption of complex B results in developmental defects. Whether these defects are unavoidable side effects or an adaptive trade-off between stress and growth remains to be determined. Also, identifying the specific RKs that partner with these SUB complexes to mediate their unique signalling outputs represents an exciting challenge for future work.

## Methods

### Plant materials and growth conditions

*Arabidopsis thaliana* (L.) Heynhold, 1842 var. Columbia (Col-0) was used as the wild-type strain. Plants were grown as previously described^22^. The *sub-9*, *sub-21*, *qky-11, chc2-1, chc2-2* and *prc1-1* mutants (Col-0) were characterized previously^17,22,24,60,76^. T-DNA insertion lines for the *NHL3* gene (*nhl3-2*, SALK_150318C; nhl3-1, SALK_035427; Col-0) are from the SALK collection and were obtained from the NASC. The lines carrying pSUB::gSUB:EGFP (in *sub-9*, Col-0), pUBQ::SUB:EGFP^31^, pUBQ10::gSUB:mCherry (Col-0), pUBQ10::GFP:QKY (in Ler) and pUBQ10::mCherry:QKY (in Ler) were previously generated^24^. Wild-type and mutant plants were transformed with different constructs using *Agrobacterium* strain GV3101/pMP90^78^ and the floral dip method^79^. Transgenic T1 plants were selected on kanamycin (50 μg ml^−1^) or hygromycin (20 μg ml^−1^) plates and viable seedlings were transferred to soil at 10 days after germination for further examination. The reporter lines pBRI1::BRI1:GFP (Col-0), pBAK1::BAK1:mCherry (Col-0), p35S::LTi6a:GFP (Col-0) and p2x35S::LTi6b:2×mCherry (Col-0) were generated as described previously^44,49,52,66,80^.

### Recombinant DNA work

Standard molecular biology techniques were used for DNA work. PCR fragments used for cloning were generated using Q5 High-Fidelity DNA Polymerase (New England Biolabs). All PCR-based constructs were sequenced by Eurofins Genomics according to their standards. pUBQ::GFP:NHL3 was assembled using the GreenGate system^81^. The complete genomic region of *NHL3* was amplified from Col-0 genomic DNA using primers gNHL3_for and gNHL3_rev. Monomeric EGFP was amplified using primers EGFP_GG_fwd and EGFP-GG_rev. Other sequences, including pUBQ10, tUBQ10, GFP and the plant resistance module, were available from the GreenGate vector library. The reporter construct was assembled into the intermediate vectors and then combined into the corresponding pGGZ0001 target vectors using a standard GreenGate reaction. To generate pGADT7/pGBKT7-NHL3 carrying the N-terminal intracellular domain, the corresponding coding region was amplified using the following primers: NdeI/NHL3_for and NHL3/SmaI_rev. Amplicons and vectors were codigested with NdeI/SmaI followed by ligation into pGADT7 and pGBKT7 vectors. The SUB intracellular domain fused to the GAL4 DNA binding domain and QKY fused to the GAL4 activation domain were described previously^24,27^. All clones were verified by sequencing.

### Co-IP and proteomics

Seedlings (7 days old; 300 mg fresh weight) expressing pSUB::SUB:EGFP were harvested and frozen in liquid nitrogen before being ground to powder using the TissueLyser II (Qiagen) unit. Ground tissue was incubated with 3 volumes of lysis buffer (50 mM Tris-Cl pH 7.5, 150 mM NaCl, 0.5 mM EDTA, 1% Triton X-100, 0.1 mM PMSF, protease inhibitor cocktail (Roche), phosphatase inhibitor cocktail (Roche) and 10% glycerol) for 10 minutes before sonicating with 5–7 pulses at 50% machine power. After centrifugation at 8,000 rpm for 15 minutes, the supernatant was incubated with prewashed GFP-Trap Magnetic Agarose (Chromotek) for 5 hours while rotating. Beads were collected magnetically and washed 3× with washing buffer (50 mM Tris-Cl pH 7.5, 150 mM NaCl, 0.1 mM PMSF, protease inhibitor cocktail (Roche), phosphatase inhibitor cocktail (Roche) and 10% glycerol) before being resuspended in 2× Laemmli buffer and boiled at 70 °C for 15 minutes to dissociate proteins from the beads. The efficiency of immunoprecipitated samples was tested by western blot with an anti-GFP antibody before being analysed by mass spectrometry. All steps were conducted at 4 °C unless stated otherwise. Immunoprecipitation eluates in LDS sample buffer (NuPAGE; Invitrogen) were reduced (10 mM dithiothreitol), alkylated (55 mM chloroacetamide) and run into a 4–12% NuPAGE gel (Invitrogen) for approximately 1 cm. In-gel digestion was performed according to standard procedures with trypsin (Roche) and TEAB digestion buffer. The generated peptides were analysed by liquid chromatography–tandem mass spectrometry on an Orbitrap HF mass spectrometer (Thermo Fisher Scientific) coupled online to a Dionex 3000 HPLC (Thermo Fisher Scientific). The liquid chromatography set-up consisted of a 75 μm × 2 cm trap column packed in-house with Reprosil Pur ODS-3 5 μm particles (Dr. Maisch) and a 75 μm × 40 cm analytical column packed with 3-μm C18 Reprosil Gold 120 particles (Dr. Maisch). Peptides were loaded onto the trap column using 0.1% FA in water. Peptides were separated on a linear gradient from 4% to 32% ACN with 5% DMSO and 0.1% formic acid in water over 60 minutes, followed by a washing step (70 minutes total method length), at a flow rate of 300 nl min^−1^ and a column temperature of 50 °C. The instrument was operated in data-dependent mode, automatically switching between MS and MS2 scans. Full-scan mass spectra (*m*/*z* 360–1,300) were acquired in profile mode with 60,000 resolution, an automatic gain control target value of 3 × 10^6^ and 10 ms maximum injection time. For the top 20 precursor ions, high-resolution MS2 scans were performed using higher-energy collisional dissociation fragmentation with 25% normalized collision energy, 30,000 resolution, an automatic gain control target value of 2 × 10^5^, 50 ms maximum injection time and 1.7 *m*/*z* isolation width in centroid mode. The underfill ratio was set to 1% with a dynamic exclusion of 20 seconds. Peptide and protein identification and quantification was performed with MaxQuant^82^ using standard settings (v.1.6.3.3). Raw files were searched against the Araport11 *Arabidopsis* protein database (Araport11_genes.201606.pep.fasta) and common contaminants. Carbamidomethylated cysteine was set as a fixed modification, and oxidation of methionine and N-terminal protein acetylation as variable modifications. Phosphorylation of serine, threonine or tyrosine were set as variable modifications. Trypsin/P was specified as the proteolytic enzyme, with up to two missed cleavages allowed. Results were filtered to 1% peptide-spectrum match, protein and site false discovery rate. All displayed protein abundances refer to MaxQuant LFQ intensities, which are based on extracted MS1 precursor ion intensities for each identified peptide feature (extracted ion chromatogram-based).

### PCR-based gene expression analysis

For quantitative real-time PCR (qPCR) of the *SUB* gene, 35–40 seedlings per biological replicate were grown for 7 days on plates under long-day conditions. RNA extraction, quality control, cDNA synthesis, qPCR and analysis were done essentially as described previously^83,84^. In brief, RNA was extracted from 7-day-old seedlings using the NucleoSpin RNA Purification Kit (Macherey-Nagel), following the manufacturer’s protocol. For qPCR, first-strand cDNA was synthesized from RNA using the RevertAid First Strand cDNA Synthesis Kit (Thermo Fisher), following the manufacturer’s protocol. qPCR was performed using the Luna Universal qPCR Master Mix (New England Biolabs) and the primers listed in Supplementary Table 1. Gene expression levels were normalized to three reference genes and analysed using the 2^−ΔΔCt^ method.

### Pharmacological treatments

Isoxaben, thaxtomin A and wortmannin were purchased from Sigma-Aldrich and used from stock solutions in DMSO (10 mM isoxaben, 2.3 mM thaxtomin A and 30 mM wortmannin). FM4-64 was purchased from Invitrogen (1.7 mM stock in water). Before imaging, 5–7-day-old *A. thaliana* seedlings were incubated for the indicated times in liquid 1/2 MS medium with 1% sucrose containing 600 nM isoxaben, 200 nM thaxtomin A, 300 mM sorbitol or 33 μM wortmannin. For FM4-64 staining, seedlings were incubated in 1 μM FM4-64 in liquid 1/2 MS medium containing 1% sucrose for 5 minutes, after which they were washed and mounted for imaging. For plasmolysis, 7–9-day-old *A. thaliana* seedlings were treated with 1 M sorbitol for 30 minutes followed by incubation with 5 μg ml^−1^ PI for 5 minutes before mounting in water for observation. The pavement cells of the cotyledons were analysed using the Olympus FV3000 set-up before images of plasmolysed protoplasts were processed and arranged using Fiji (ImageJ) v.1.54p^85^.

### Lignin, callose and ROS staining

Stratified seeds were grown vertically on a 10° slope on square plates containing 1/2 MS medium and 1% sucrose supplemented with 0.9% agar for 7 days at 22 °C under continuous light. Seedlings (7 days old) were then transferred to multiwell plates containing 1/2 MS liquid medium supplemented with 1% sucrose and incubated overnight for lignin and ROS, and 24 hours for callose. The medium was then replaced with fresh 1/2 MS liquid medium containing either mock (DMSO) or 600 nM isoxaben, and seedlings were incubated for the indicated time. Staining for lignin (phloroglucinol), callose (aniline blue) and ROS (H2DCFDA) was performed as previously described^16,17,86^. In brief, lignification was assessed using phloroglucinol-HCl staining, while callose deposition was visualized by destaining cotyledons in acetic acid–ethanol followed by staining with 0.01% aniline blue for at least 2 hours in the dark. ROS staining was performed by incubating seedlings in liquid medium supplemented with 100 μM H2DCFDA for 10 minutes in the dark before each time point. Lignified root images were captured using a Leica SAPO stereomicroscope and Leica MC170HD camera. Aniline blue-stained cotyledons were imaged using an Olympus FV1000 set-up under a 10× objective. ROS accumulation was analysed using an Olympus FV3000 set-up under a 20× objective. For quantification, a defined region of interest (ROI) located 300 µm above the root tip (excluding the root cap) was used in lignified samples. ROS accumulation was determined by measuring overall ROS signal in the depicted root region. Micrographs with strong aniline blue signal excluding all leaf veins were used for callose analysis. Quantification was performed in Fiji (ImageJ) v.1.54p.

### Y2H assay

The Matchmaker Y2H system (Takara Bio Europe) was used and experimental procedures followed the manufacturer’s recommendations. Constructs of interest fused to the prey vector pGADT7 with the Gal4 activation domain and to the bait vector pGBKT7 with the Gal4 DNA-binding domain were co-transformed into the yeast strain AH109. Transformants were selected on synthetic drop-out medium lacking leucine and tryptophan (-LW) at 30 °C for 2–3 days. To examine direct interactions, transformants were streaked out of drop-out medium plates lacking leucine, tryptophan and histidine (-LWH). Standard -LWH growth medium was supplemented with 2.5 mM 3-amino-1,2,4-triazole to minimize false positive signals. Yeast was grown at 30 °C for an additional 2–3 days.

### Quantification of fungal DNA by qPCR

To quantify the fungal DNA, infected seedlings were harvested at 5 days post-infection before DNA extraction, in accordance with standard protocols. qPCR experiments were conducted using a Maxima SYBR green mix (Thermo Fisher Scientific) on an Agilent AriaMx Real-Time qPCR system (Agilent Technologies). The quantity of fungal DNA was calculated in relation to the amount of plant DNA. This was achieved by using primers for the At*RbcS* (*Arabidopsis* small RuBisCO subunit) and *E. cruciferarum* tubulin genes (Supplementary Table 1).

### Microscopy

Rosettes and siliques were imaged using a Leica SAPO stereomicroscope equipped with an MC 170 HD digital camera (Leica Microsystems). Clearing and imaging of ovules was performed as previously described^87^. A 10× objective was used for root hair pattern analysis. The number of cells in H-position (trichoblasts) and N-position (atrichoblasts) in at least eight seedlings was counted, and the relative ratios of H-position cells with root hairs and N-position cells without root hairs were calculated. Confocal laser scanning microscopy was performed using an Olympus FV3000 set-up with a high-sensitivity detector and a 60× water immersion objective (1.2 NA; Olympus Europa). The scanning speed was set to 4.0 μs pixel^−1^ (image size 512 × 512 pixels) and the line average was set to 10, with a digital zoom of up to 4×. For subcellular localization of SUB–EGFP and GFP–NHL3, confocal laser scanning microscopy was performed on epidermal cells of root meristems located approximately 100 µm above the quiescent centre and on pavement cells of 6–8-day-old cotyledons of *A. thaliana* seedlings. GFP fluorescence was excited at 488 nm with a diode laser (3% intensity) and emission was detected at 500–540 nm at 4× digital zoom. The mCherry fluorescence, FM4-64 plasma membrane marker and PI were excited with a 561 nm laser (3–5% intensity) and emission was detected at 584–653 nm. Aniline blue fluorescence was excited with a 405 nm laser (1% intensity) and detected at 425–475 nm. H2DCFDA fluorescence was excited with a 488 nm laser (1% intensity). To determine colocalization, the distance from the centre of each GFP spot to the centre of the nearest FM4-64 signal was measured manually on single optical sections using Fiji (ImageJ) v.1.54p^85^. If the distance between 2 puncta was less than the resolution limit of the objective lens (0.24 µm), the signals were considered to colocalize^67^. For direct comparisons of fluorescence intensities, the laser, pinhole and gain settings of the confocal microscope were kept identical when imaging seedlings expressing SUB–EGFP in different mutant backgrounds. The intensities of fluorescence signals at the plasma membrane were quantified using Fiji (ImageJ) v.1.54p. To examine the fluorescence levels at the plasma membrane, ROIs covering only the plasma membrane of six to ten single cells from each seedling were used for measurements. The mean pixel intensities for the selected ROIs were recorded, normalized to the corresponding background, and the corrected total cell fluorescence was calculated. For analysis of ROS accumulation, the whole-root signal was used for measurements and the corrected total cell fluorescence was calculated. *Arabidopsis* seedlings were covered with a 22 mm × 22 mm glass coverslip with a thickness of 0.17 mm (no. 1.5H; Paul Marienfeld). Images were adjusted for colour and contrast using Fiji (ImageJ) v.1.54p.

### FRET-FLIM and FA measurements

FRET-FLIM was performed using an Olympus FV3000 microscope equipped with a time-correlated single photon-counting (TCSPC) device (LSM upgrade kit; PicoQuant). Root epidermal cells of 5-day-old seedlings expressing GFP fusion protein alone or coexpressing GFP–mCherry fusion protein pairs of interest were imaged using a 60× water immersion objective (1.2 NA) at 4× digital zoom. The scanning speed was 4.0 μs pixel^−1^ (image size 512 × 512 pixels). The FLIM filter cube DIC560 for GFP/RFP was used. GFP fluorescence lifetimes were measured using two photon-counting PMA Hybrid 40 detectors and a pulsed 485 nm diode laser (LDH-D-C-485; PicoQuant) at a laser pulse rate of 40 MHz. A minimum of 100,000 photons were acquired for each image with a TCSPC resolution of 25.0 ps (image size 512 × 512 pixels). Results were analysed using SymPhoTime 64 software 2.11 (PicoQuant) with n-exponential reconvolution and an internally calculated instrument response function. The lifetime fitting model parameter was used up to *n* = 2. Analysis results with a correction factor (*χ*^2^) between 0.9 and 2.0 were accepted. The intensity weighted average lifetime (τ Av_Int) of the ROIs was taken as the final value for each image. Steady-state FA was performed and analysed as previously reported^13,88^.

### Statistical analysis

Statistical analysis was performed using PRISM 10.6.1 software (GraphPad Software). All statistical tests and *P* values are described in the respective figure legends. For the box-and-whisker plots, whisker ends mark the minimum and maximum values, and the horizontal line marks the mean of all data. Experiments were repeated the number of times indicated in each figure legend. All replication attempts were successful.

### Reporting summary

Further information on research design is available in the Nature Portfolio Reporting Summary linked to this article.

## Supplementary information

Supplementary Information Supplementary Table 1

Reporting Summary

Peer Review File

## Source data

Source Data Figs. 1–5, Extended Data Figs. 1–7 and Extended Data Table 1 Source data.

## Data Availability

All relevant data and materials are available from the corresponding author upon request. The original data points in graphs and statistical analyses are provided in the Source data.

## Extended data

**Extended Data Table 1:** Distribution of root hair and nonhair cells in the root epidermis

Genotype	n(roots)	Cells total	H position		Cells total	N position		
			Hair (%)	Non-hair (%)		Hair (%)	Non-hair(%)	
Col-0	16	837	98.95±1.81	1.05±1.81	996	0.55±1.28	99.45±1.28	
sub-9*	16	696	85.47±5.75	14.53±5.75	759	13.86±3.83	86.14±3.83	
nhl3-2	16	792	99.11±1.24	0.89±1.24	887	1.52±1.83	98.48±1.83	

Data are mean ± s.d.

*Tukey’s multiple comparisons tests revealed significant differences (P < 0.0001) for the following comparisons: Col versus *sub-9* and *sub-9* versus *nhl3-2*. The experiment was repeated twice with similar results. Statistical parameters and exact P values are shown in the Source data file.

Source data
