# Supplementary material for: A STRUBBELIG–NHL3 cell surface receptor complex mediates the response to cellulose deficiency in Arabidopsis
Source: Nat Plants. 2026 Aug 14;12(8):1514–27. doi: 10.1038/s41477-026-02342-4 (PMC13481253; doi:10.1038/s41477-026-02342-4)
Supplement: Supplementary file 1 [file 41477_2026_2342_MOESM1_ESM.pdf]

nature plants

Supplementary information https://doi.org/10.1038/s41477-026-02342-4

A STRUBBELIG–NHL3 cell surface receptor complex mediates the response to cellulose deficiency in *Arabidopsis*

In the format provided by the authors and unedited

Primer name	Sequence (5'- 3')
gNHL2_for	AACAGGTCTCAGGCTCAACAATGGGGTCCAAACAACCATA
gNHL2_rev	AACAGGTCTCTCTGAAGATAAGTCAAAGTCACACT
gNHL3_for	GCTTGGTCTCAGGCTCAACAATGGCGGACTTAAACGGTGCGT
gNHL3_rev	ATTCGGTCTCACTGAAAAGTCAACGTCACACTTGGTC
NdeI/NHL2_for	GGAATTCCATATGTTCCGTCCAAACGCCG
NHL2/SmaI_rev	TCCCCCGGGCTAAGATAAGTCAAAGTCACACTGC
NdeI/NHL3_for	GGAATTCCATATGAGACCCAACGCTATCAAATTC
NHL3/SmaI_rev	TCCCCCGGGAAAGTCAACGTCACACTTGGT
sgRNA1	CCTAAGGCTCACCGCAGCTA
sgRNA2	CATAGCTGTAGCCGTAATCC
