# Supplementary material for: A STRUBBELIG–NHL3 cell surface receptor complex mediates the response to cellulose deficiency in Arabidopsis
Source: Nat Plants. 2026 Aug 14;12(8):1514–27. doi: 10.1038/s41477-026-02342-4 (PMC13481253; doi:10.1038/s41477-026-02342-4)
Supplement: Supplementary file 2 [file 41477_2026_2342_MOESM2_ESM.pdf]

nature portfolio

Corresponding author(s): Kay Schneitz

Last updated by author(s): June 08, 2026

Reporting Summary

Nature Portfolio wishes to improve the reproducibility of the work that we publish. This form provides structure for consistency and transparency in reporting. For further information on Nature Portfolio policies, see our Editorial Policies and the Editorial Policy Checklist.

Statistics

For all statistical analyses, confirm that the following items are present in the figure legend, table legend, main text, or Methods section.

n/a	Confirmed	
☐	☒	The exact sample size (*n*) for each experimental group/condition, given as a discrete number and unit of measurement
☐	☒	A statement on whether measurements were taken from distinct samples or whether the same sample was measured repeatedly
☐	☒	The statistical test(s) used AND whether they are one- or two-sided<br>*Only common tests should be described solely by name; describe more complex techniques in the Methods section.*
☐	☒	A description of all covariates tested
☒	☐	A description of any assumptions or corrections, such as tests of normality and adjustment for multiple comparisons
☐	☒	A full description of the statistical parameters including central tendency (e.g. means) or other basic estimates (e.g. regression coefficient) AND variation (e.g. standard deviation) or associated estimates of uncertainty (e.g. confidence intervals)
☐	☒	For null hypothesis testing, the test statistic (e.g. *F*, *t*, *r*) with confidence intervals, effect sizes, degrees of freedom and *P* value noted<br>*Give P values as exact values whenever suitable.*
☒	☐	For Bayesian analysis, information on the choice of priors and Markov chain Monte Carlo settings
☒	☐	For hierarchical and complex designs, identification of the appropriate level for tests and full reporting of outcomes
☒	☐	Estimates of effect sizes (e.g. Cohen's *d*, Pearson's *r*), indicating how they were calculated

Our web collection on statistics for biologists contains articles on many of the points above.

Software and code

Policy information about availability of computer code

Data collection	Fluoview Olympus3000 (Software: Olympus FV 3000 Imaging Software, CellSens Desktop Version 1) was used for confocal microscopy and PicoQuant advanced FCS/FLIM-FRET/rapidFLIM upgrade kit (Software: SymPhoTime64 2.11) for FRET-FLIM and fluorescence anisotropy. Leica TCS SP8 X WLL2 upright HyVolution 2 (Software: LAS X SP8 Control Software/Dye Finder/Live Data Mode/MicroLab) for morphological analysis. Leica MC170 HD 5 MP HD Microscope Camera (Software: LAS EZ 3.4 DVD 272) for phloroglucinol staining.
Data analysis	PicoQuant advanced FCS/FLIM-FRET/rapidFLIM upgrade kit (Software: SymPhoTime64) for FRET-FLIM and fluorescence anisotropy analysation. Fiji/ImageJ 1.54p for confocal micrograph and lignin analyzation.

For manuscripts utilizing custom algorithms or software that are central to the research but not yet described in published literature, software must be made available to editors and reviewers. We strongly encourage code deposition in a community repository (e.g. GitHub). See the Nature Portfolio guidelines for submitting code & software for further information.

Data

Policy information about availability of data

All manuscripts must include a data availability statement. This statement should provide the following information, where applicable:

- Accession codes, unique identifiers, or web links for publicly available datasets
- A description of any restrictions on data availability
- For clinical datasets or third party data, please ensure that the statement adheres to our policy

Identifiers for published or publicly available lines are provided in the Material and Methods. Any other relevant biological materials used in this study are available from the corresponding author upon request.

Research involving human participants, their data, or biological material

Policy information about studies with human participants or human data. See also policy information about sex, gender (identity/presentation), and sexual orientation and race, ethnicity and racism.

Reporting on sex and gender	not applicable
Reporting on race, ethnicity, or other socially relevant groupings	not applicable
Population characteristics	not applicable
Recruitment	not applicable
Ethics oversight	not applicable

Note that full information on the approval of the study protocol must also be provided in the manuscript.

Field-specific reporting

Please select the one below that is the best fit for your research. If you are not sure, read the appropriate sections before making your selection.

☒ Life sciences ☐ Behavioural & social sciences ☐ Ecological, evolutionary & environmental sciences

For a reference copy of the document with all sections, see nature.com/documents/nr-reporting-summary-flat.pdf

Life sciences study design

All studies must disclose on these points even when the disclosure is negative.

Sample size	Sample sizes (n) were chosen based on standard practice in the literature and prior experience, reflecting the number of independent biological replicates measured per experiment.
Data exclusions	Data were solely excluded based on statistical outlier detection using ESD method. All other data points are included.
Replication	One objective of this study was to ensure reproducibility. This was achieved by conducting a minimum of two sets of experiments, though three sets were conducted in most cases. Plasmolysis comprises one set of experiments.
Randomization	Samples were randomly assigned to experimental groups
Blinding	Blinding was not applied as measurements were fully automated and objective, with identical analysis settings applied to all samples

Reporting for specific materials, systems and methods

We require information from authors about some types of materials, experimental systems and methods used in many studies. Here, indicate whether each material, system or method listed is relevant to your study. If you are not sure if a list item applies to your research, read the appropriate section before selecting a response.

Materials & experimental systems

n/a	Involved in the study
☒	☐ Antibodies
☒	☐ Eukaryotic cell lines
☒	☐ Palaeontology and archaeology
☒	☐ Animals and other organisms
☒	☐ Clinical data
☒	☐ Dual use research of concern
☐	☒ Plants

Methods

n/a	Involved in the study
☒	☐ ChIP-seq
☒	☐ Flow cytometry
☒	☐ MRI-based neuroimaging

Plants

Seed stocks	Arabidopsis thaliana seed stocks used in this study have been described previously (Material & Methods). T-DNA insertion lines for the NHL3 gene (nhl3-2, SALK_150318C; nhl3-1, SALK_035427; Col-0) are from the SALK collection and were obtained from the NASC.
Novel plant genotypes	The pUBQ::GFP:NHL3 reporter construct was assembled using the GreenGate system. Wild-type and mutant plants were transformed using Agrobacterium strain GV3101/pMP90 73 and the floral dip method. Transgenic T1 plants were selected on selective plates and viable seedlings were transferred to soil.
Authentication	Most seed stocks used were previously described (Material & Methods), and their identity was verified by reference to published genotypes or phenotypes. Novel or modified lines were genotyped using PCR to confirm the presence of relevant alleles. Transgenic lines were verified by functional complementation of the corresponding mutants.
