# Supplementary material for: A STRUBBELIG–NHL3 cell surface receptor complex mediates the response to cellulose deficiency in Arabidopsis
Source: Nat Plants. 2026 Aug 14;12(8):1514–27. doi: 10.1038/s41477-026-02342-4 (PMC13481253; doi:10.1038/s41477-026-02342-4)
Supplement: Supplementary file 3 [file 41477_2026_2342_MOESM3_ESM.pdf]

nature portfolio

Peer Review File

A STRUBBELIG-NHL3 cell surface receptor complex mediates the response to cellulose deficiency in Arabidopsis

Corresponding Author: Professor Kay Schneitz

Version 0:

Reviewer comments:

Reviewer #1

(Comments for the Author)
Boikine et al. show in this manuscript how the recently established new player in cell wall integrity stress, the receptor kinase STRUBBELIG (SUB), could mediate the reaction to cellulose biosynthesis inhibition (CBI). The authors identify NHL3 as a direct interactor of SUB and show their joint role in the Arabidopsis response to CBI. The authors propose that SUB acts as a co-receptor forming several complexes (SUB–NHL3, SUB–NHL3–QKY, and SUB–QKY) that regulate distinct aspects of the CBI response or different developmental responses. The data demonstrate a clear physical interaction between SUB and NHL3, and functional assays show that both are required for normal callose and lignin deposition following cellulose perturbation. Very interestingly, the authors also propose a new time frame for some responses to CBI, such as SUB internalization, which begins within 30 minutes rather than 4–5 hours as previously thought. The study is technically competent and addresses a relevant topic in cell-wall integrity signalling. However, I have some concerns regarding the proposed mechanistic model and interpretation of key findings:
1. Evidence for a ternary complex and its biochemical basis. In the discussion (lines 400–408) the authors propose three distinct SUB-containing complexes, including complex B (SUB–NHL3–QKY) that would specifically regulate lignification. However, the biochemical support for this ternary complex is lacking. NHL3 and QKY cannot directly interact (NHL3–QKY interaction is negative, Supplementary Fig. 4, lines 142–145). Additionally, NHL3, unlike QKY, does not promote SUB homo-oligomerization (lines 271–272: "minimal difference in FA values indicates that NHL3...does not promote SUB homo-multimerization"). These observations suggest that NHL3 and QKY have fundamentally different biochemical roles. The existence of a ternary complex is therefore inferred from overlapping phenotypes, not shown biochemically. The authors acknowledge that NHL3 and QKY function in parallel (lines 284–285) and propose that "additional SUB complexes containing NHL3 or QKY that are not subject to CBI-induced internalization are likely also present" (lines 412–414). This raises a critical question: if NHL3 and QKY have distinct oligomerization properties and cannot directly interact, what is the molecular basis for postulating they assemble into the same ternary complex? The model would be stronger if the authors were more explicit about whether complex B represents a simultaneous trimeric assembly or a temporal succession of binary complexes on the same receptor, as stated in the Abstract. The model shown in fig S7 should be revised accordingly and more emphasis should be given to complexes A and C which are well-supported.
2. Interpretations of some interactions could be improved from the mechanistic point of view. For example, the temporal dissociation of SUB interactions with differential kinetics. The authors report that isoxaben rapidly dissociates SUB–NHL3 interaction (undetectable by 30 minutes, lines 322–323), while SUB–QKY interaction remains prominent at 30 minutes (line 325) and only shows substantial attenuation after 5 hours (lines 326–330). This marked temporal difference (15 min vs. 5 hours, ~200-fold difference in timescale) is striking and raises an important mechanistic question: what explains this dramatic difference in the kinetics of dissociation? The paper shows that both interactions are disrupted by CBI, but the molecular basis for this temporal hierarchy is not explained. The authors propose (lines 356–357) that "a likely cell-wall-derived signal" rapidly activates SUB, but provide no mechanistic explanation for why this signal would release NHL3 rapidly while leaving QKY relatively stable for hours.
3. The authors report that SUB–NHL3 interaction decreases within 15 minutes of isoxaben treatment (line 322) and SUB internalization increases within 30 minutes (lines 295–297). Yet lignification, a late CBI response measured at 16 hours (lines 164–165), remains SUB-dependent (lines 165–167). This timing raises an important mechanistic question: does SUB signaling require continuous plasma membrane presence, does endosomal localization enhance activation of the pathway (like in the case of BRI1)? Or is SUB is recycled back to the membrane?
Suggestions listed below mainly address the readability of the paper. Although very clearly written in general, I think some parts of this paper could be improved.
Fig S1a legend – please briefly explain how the protein abundance is measured.
Fig S2b and other box-plots figs – please explain what is presented as the box including its horizontal line, possibly in one common sentence for all parts of the figures.
Fig S3c,d,e – please briefly explain that these are FRET/FLIM experiments and that the lifetime reduction indicates an interaction at the first mention of this technique. In this fig and other similar figs, please state what represents “n”, number of

plants?
L 91. Please state the full name of LTi6b and indicate if it is an Arabidopsis protein
L130 – the part of the sentence directly after "or" – redundant?
L 173. The claim of similar effects in case of callose induction for the different mutants should be narrowed as not all mutants were tested for callose induction as shown in Fig. S7 and qky mutants were previously reported not to affect this response.
Fig. 3hi – I presume TMO7:GPF constructs were used as a positive control. This should be explained. The identity of TMO7-encoded protein should be stated and the choice of this reporter explained. The attention of the reader should be pointed to the result concerning SBE reporter in the figure legend.
L277. "The available data suggest" – please point to these data.
Fig 4. The layout of this fig could be improved to make it similar to other figs where the confocal images are followed by fluorescence quantification, thus C after D.
L275-331. I find the presentation of data shown in Fig 4, difficult to follow because the narrative does not follow the order of sub-panels in this fig.
Fig S7 – could the revised model figure be part of the main figs?
Overall, this is a well-conceived and technically sound study that adds valuable information to the field of receptor-mediated cell wall integrity responses, and I would like to congratulate the authors for it.

Reviewer #2

(Comments for the Author)
Proper intracellular responses to cell wall damage or remodeling are important for plants to maintain normal growth and sustain resistance to environmental stresses. Plasma membrane-localized receptor-like kinases play a key role in transducing cell wall signals, among which SUB is an important one that responds to cellulose biosynthesis inhibition. In this study, the authors identified NHL2 and NHL3 as interacting partners of SUB. Genetic data showed that nhl2 and nhl3 mutants exhibited defects in lignin deposition triggered by isoxaben treatment, similar to the sub mutant, suggesting they may function in the same pathway. Further study showed that NHL3 is required for maintaining the abundance of SUB at the plasma membrane. However, the underlying mechanism remains unclear. Overall, this study is relatively preliminary and lacks mechanistic insights into how SUB and NHL2/3 regulate each other. Additionally, some of the results are not robust enough to support the conclusions. The major points are as follows:

1. In Fig. S3, to confirm the plasma membrane localization of NHL2-GFP and NHL3-GFP, a membrane fractionation assay combined with immunoblotting should be performed.
2. In Fig. 2, the self-activation of NHL3 should be examined in the Y2H assay.
3. Co-IP assays must be performed to validate the interaction between NHL3 and SUB.
4. Fig. 3c does not show the staining images for sub-9, sub-21, nhl2-1, and nhl3-2.
5. Fig. 3c and 3d, how to explain that NHL2 does not interact with SUB, but nhl2-1 single mutant exhibited a similar defect as sub in lignin deposition triggered by isoxaben? What is the relationship between NHL2 and NHL3? How to explain that nhl2-1 nhl3-2 double mutant exhibited a severity of lignin deposition similar to the single mutants?
6. The resolution of the images in Fig. S9 is extremely low.
7. In Fig. 3a, the transcript levels of SUB:EGFP in each genotype should be examined before examining the fluorescence signals. To confirm the reduction of SUB:EGFP at the plasma membrane in nhl3-2 and qky-11, membrane fractionation assays are recommended. Given that nhl2 did not affect the localization of SUB:EGFP to the plasma membrane, how to explain that nhl2 exhibited a similar defect as sub and nhl3 in lignin deposition?
8. In Fig. 4a, the difference in SUB:EGFP localization at the plasma membrane between wild-type, nhl3-2, and qky-11 backgrounds is minimal. Membrane fractionation assays are strongly recommended to validate these results.
9. The authors should investigate the molecular mechanisms by which NHL2 and NHL3 promote the plasma membrane localization of SUB:EGFP.
10. As a kinase, whether SUB phosphorylates NHL2 and NHL3 warrants further investigation.

Reviewer #3

(Comments for the Author)
The article by Boikine et al describe the function of STRUBBELIG (SUB) receptor kinase in the regulation of cellular responses associated to cell wall integrity (CWI) alteration triggered by treatment of plants with the cellulose biosynthesis inhibitors (CBI). The authors discovered that NHL2 and NHL3, two proteins that have been involved previously in plant response to stresses, are additional factors involved in the regulation of CBI response. These findings increase the number of proteins involved in CWI regulation mediated by CIB, with SUB receptor kinase, preciously described by the group, playing a key role. Authors, also tested the genetic and cellular interactions between these SUB and NHL2/NHL3 components. The article data show that the function NHL3 on cell wall integrity control is dependent of SUB, and the article also contains novel sets of biochemically and functionally distinct SUB complexes involved in regulating cell wall integrity and development. The microscopic and genetic analyses performed are of great quality and the characterization of the different complexes identified at the membrane/cellular levels are well documented. The article contains interesting and novel information and a very well characterization of SUB interaction with and NHL3 at the cellular level. However, some of the conclusions of the article are not supported by the data. Also, a more detailed characterization of the functional mechanism of SUB/NHL3 and NHL2 to explain CBI triggered cell wall integrity alterations would be needed. Last, a clear link between cell wall integrity modification and the activation of SUB/NHL3 and NHL2 has not been shown.

Comments
1. The phenotypic determination of alteration of CWI by CBI in the article is just based on the measurement of lignin accumulation in the transition/elongation zone of the root tip and callose production in the cotyledons, that are clearly two molecules that are deposited in the cell walls. However, additional analyses of cell wall integrity alterations (biochemical determination, immunodetection of changes in specific cell wall components (e.g. pectin), etc.) were not performed. A more detailed characterization of cell wall integrity alterations would be needed to distinguish, for example, among NHL2 and NHL3 (dependent on SUB) mediated mechanisms observed and to further support some of the conclusion of the article (e.g. pectin changes, see point 6). Though some data were generated with mutants defective in CESAs required for primary cell wall biosynthesis, these analyses are based on genetics.
2. The article nicely shows that the read-out of CWI alteration triggered by CIB require NHL2 and NHL3, based on the analyses of different set of mutants (single and higher order) since lignin dose not accumulate in nhl mutants. Interestingly, overexpression of SUB rescues the nhl3-2 mutant phenotype. Also. it is shown that CBI disrupts the physical interaction of SUB with NHL3 and QKY. However, the impact of all these interaction disruptions at the regulatory level (downstream signalling, regulation of genes, etc.) have not been determined. For example, what are the differences at the regulatory level between NHL2 and NHL3 activation of CWI mediated responses? Molecular bases of these different mechanisms should be determined, since the readout (lignin accumulation) is identical, but the pathways seem to be different.
3. It has not been characterised how CBI mediates SUB internalization or blocks SUB homo-oligomerization. What is the signal for SUB internalization?
4. Authors suggest that NHL3 is a more general stress response factor than previously believed. NHL3 has been previously shown to participate in cell wall remodelling induced by the perception of pathogens. Whether the mechanism regulated by NHL3 in pathogen perception and CBI-induced cell wall damage regulation share some components or are specific processes has not been determined.
5. Authors suggest that application of isoxaben or thaxtomin A first triggers a likely cell wall-derived signal that rapidly activates SUB signalling, but not very well know cell wall derived DAMPs have been tested experimentally in the experiments performed.
6. Authors propose that CBI-induced changes in the SUB/QKY complex architecture are due to cell wall signalling responding to such secondary alterations in the cell wall, possibly related to pectin, but changes in pectin composition/structure in cell walls upon CIB have not been tested/determined. Can be the phenotypes observed in sub and hhl3 mutants mimicked by adding exogenously OGs or cellulose-derived DAMPs?
7. NHL2 and NHL3 are not involved in SUB-dependent plant development. Explanation for this?. Is SUB involve in disease resistance?

Decision Letter:

25th November 2025

Dear Professor Schneitz,

Your Article, "A STRUBBELIG-NHL3 cell surface receptor complex mediates the response to cellulose deficiency in Arabidopsis" has now been seen by three referees. You will see from their comments below that while they find your work of interest, some important points are raised. We are interested in the possibility of publishing your study in Nature Plants, but would like to consider your response to these concerns in the form of a revised manuscript before we make a final decision on publication.

As you can read below, all reviewers think that the findings are potentially interesting and relevant, however, more evidence is required to fully support the main conclusions. In our opinion the main points are:

1. Reviewer #1 thinks that biochemical support for the ternary SUB–NHL3–QKY complex is lacking, and that observations rather suggest that NHL3 and QKY have fundamentally different biochemical roles. Furthermore, it is unclear whether SUB signaling requires continuous plasma membrane presence or is enhanced by endosomal localization.
2. Reviewer #2 states that further assays (e.g. fractionation, Y2H, CoIP) are required to support some of the conclusions. Furthermore, the mechanism by which NHL2/3 promote PM localization of SUB and whether SUB can phosphorylate NHL2/3 should be examined.
3. Reviewer #3 thinks that a more detailed characterization of cell wall integrity alterations is needed to support some of the conclusions. The impact of variations in SUB-NHL2/3-QKY interactions should be analyzed downstream on the level of signaling and gene regulation.

All referees also raise some other points that should be addressed. As usual, please do not hesitate to get in touch if you would like to discuss these issues further.

We therefore invite you to revise your manuscript taking into account all reviewer and editor comments. Please highlight all changes in the manuscript text file, preferably in Microsoft Word format.

We are committed to providing a fair and constructive peer-review process. Do not hesitate to contact us if there are specific requests from the reviewers that you believe are technically impossible or unlikely to yield a meaningful outcome.

When revising your manuscript please:

* Include a "Response to referees" document detailing, point-by-point, how you addressed each referee comment. If no action was taken to address a point, you must provide a compelling argument. This response will be sent back to the referees along with the revised manuscript.

* If you have not done so already please begin to revise your manuscript so that it conforms to our Article format instructions at http://www.nature.com/nplants/info/final-submission. Refer also to any guidelines provided in this letter.

* Include a revised version of any required reporting checklist. It will be available to referees (and, potentially, statisticians) to aid in their evaluation if the manuscript goes back for peer review. A revised checklist is essential for re-review of the paper.

* Pay close attention to our href="https://www.nature.com/nature-portfolio/editorial-policies/image-integrity">Digital Image Integrity Guidelines. and to the following points. Please ensure:
-- that unprocessed scans are clearly labelled and match the gels and western blots presented in figures.
-- that control panels for gels and western blots are appropriately described as loading on sample processing controls
-- that all images in the paper are checked for duplication of panels and for splicing of gel lanes.
-- that you retain unprocessed data and metadata files after publication, ideally archiving data in perpetuity. These may be requested during the peer review and production process or after publication if any issues arise.

EXTENDED DATA FIGURES

When re-submitting your manuscript, please ensure that any supplementary figures and tables that are crucial to the manuscript's conclusions are converted into Extended Data figures and tables to increase visibility of these data. Extended Data figures and tables are online-only (present in the online PDF and full-text HTML versions of the paper), peer-reviewed display items that provide essential background to the article but are not included in the main article due to space constraints. A maximum of ten Extended Data display items (figures and tables) is permitted.

Nature Plants strongly supports public availability of data and are therefore keen that the data used in your paper is placed in an appropriate public data repository. Alternatively, if this is not possible, you may present the data as Extended Data or Supplementary Information. If data can only be shared on request, please explain why in your Data Availability Statement, and also in the correspondence with your editor. Please note that for some data types, deposition in a public repository is mandatory.

Please use the link below to submit your revised manuscript and related files:

Link Redacted

Note: This URL links to your confidential home page and associated information about manuscripts you may have submitted, or that you are reviewing for us. If you wish to forward this email to co-authors, please delete the link to your homepage.

We hope to receive your revised manuscript within four to five months, however, this is not a strict deadline. If you cannot send it within this time, please let us know. We will be happy to consider your revision so long as nothing similar has been accepted for publication at Nature Plants or published elsewhere.

We look forward to seeing the revised manuscript and thank you for the opportunity to review your work.

Sincerely,

Nature Plants is committed to improving transparency in authorship. As part of our efforts in this direction, we are now requesting that all authors identified as 'corresponding author' on published papers create and link their Open Researcher and Contributor Identifier (ORCID) with their account on the Manuscript Tracking System (MTS), prior to acceptance. This applies to primary research papers only. ORCID helps the scientific community achieve unambiguous attribution of all scholarly contributions. You can create and link your ORCID from the home page of the MTS by clicking on 'Modify my Springer Nature account'. For more information please visit please visit www.springernature.com/orcid.

Please do not hesitate to contact me if you have any questions or would like to discuss these revisions further.

****

Reviewers' Comments:

Reviewer #1 (Comments for the Author):

Boikine et al. show in this manuscript how the recently established new player in cell wall integrity stress, the receptor kinase STRUBBELIG (SUB), could mediate the reaction to cellulose biosynthesis inhibition (CBI). The authors identify NHL3 as a direct interactor of SUB and show their joint role in the Arabidopsis response to CBI. The authors propose that SUB acts as a co-receptor forming several complexes (SUB–NHL3, SUB–NHL3–QKY, and SUB–QKY) that regulate distinct aspects of the

CBI response or different developmental responses. The data demonstrate a clear physical interaction between SUB and NHL3, and functional assays show that both are required for normal callose and lignin deposition following cellulose perturbation. Very interestingly, the authors also propose a new time frame for some responses to CBI, such as SUB internalization, which begins within 30 minutes rather than 4–5 hours as previously thought. The study is technically competent and addresses a relevant topic in cell-wall integrity signalling. However, I have some concerns regarding the proposed mechanistic model and interpretation of key findings:

1. Evidence for a ternary complex and its biochemical basis. In the discussion (lines 400–408) the authors propose three distinct SUB-containing complexes, including complex B (SUB–NHL3–QKY) that would specifically regulate lignification. However, the biochemical support for this ternary complex is lacking. NHL3 and QKY cannot directly interact (NHL3–QKY interaction is negative, Supplementary Fig. 4, lines 142–145). Additionally, NHL3, unlike QKY, does not promote SUB homo-oligomerization (lines 271–272: "minimal difference in FA values indicates that NHL3...does not promote SUB homo-multimerization"). These observations suggest that NHL3 and QKY have fundamentally different biochemical roles. The existence of a ternary complex is therefore inferred from overlapping phenotypes, not shown biochemically. The authors acknowledge that NHL3 and QKY function in parallel (lines 284–285) and propose that "additional SUB complexes containing NHL3 or QKY that are not subject to CBI-induced internalization are likely also present" (lines 412–414). This raises a critical question: if NHL3 and QKY have distinct oligomerization properties and cannot directly interact, what is the molecular basis for postulating they assemble into the same ternary complex? The model would be stronger if the authors were more explicit about whether complex B represents a simultaneous trimeric assembly or a temporal succession of binary complexes on the same receptor, as stated in the Abstract. The model shown in fig S7 should be revised accordingly and more emphasis should be given to complexes A and C which are well-supported.

2. Interpretations of some interactions could be improved from the mechanistic point of view. For example, the temporal dissociation of SUB interactions with differential kinetics. The authors report that isoxaben rapidly dissociates SUB–NHL3 interaction (undetectable by 30 minutes, lines 322–323), while SUB–QKY interaction remains prominent at 30 minutes (line 325) and only shows substantial attenuation after 5 hours (lines 326–330). This marked temporal difference (15 min vs. 5 hours, ~200-fold difference in timescale) is striking and raises an important mechanistic question: what explains this dramatic difference in the kinetics of dissociation? The paper shows that both interactions are disrupted by CBI, but the molecular basis for this temporal hierarchy is not explained. The authors propose (lines 356–357) that "a likely cell-wall-derived signal" rapidly activates SUB, but provide no mechanistic explanation for why this signal would release NHL3 rapidly while leaving QKY relatively stable for hours.

3. The authors report that SUB–NHL3 interaction decreases within 15 minutes of isoxaben treatment (line 322) and SUB internalization increases within 30 minutes (lines 295–297). Yet lignification, a late CBI response measured at 16 hours (lines 164–165), remains SUB-dependent (lines 165–167). This timing raises an important mechanistic question: does SUB signaling require continuous plasma membrane presence, does endosomal localization enhance activation of the pathway (like in the case of BRI1)? Or is SUB is recycled back to the membrane?

Suggestions listed below mainly address the readability of the paper. Although very clearly written in general, I think some parts of this paper could be improved.

Fig S1a legend – please briefly explain how the protein abundance is measured.

Fig S2b and other box-plots figs – please explain what is presented as the box including its horizontal line, possibly in one common sentence for all parts of the figures.

Fig S3c,d,e – please briefly explain that these are FRET/FLIM experiments and that the lifetime reduction indicates an interaction at the first mention of this technique. In this fig and other similar figs, please state what represents “n”, number of plants?

L 91. Please state the full name of LTi6b and indicate if it is an Arabidopsis protein

L130 – the part of the sentence directly after “or” – redundant?

L 173. The claim of similar effects in case of callose induction for the different mutants should be narrowed as not all mutants were tested for callose induction as shown in Fig. S7 and qky mutants were previously reported not to affect this response.

Fig. 3hi – I presume TMO7:GPF constructs were used as a positive control. This should be explained. The identity of TMO7-encoded protein should be stated and the choice of this reporter explained. The attention of the reader should be pointed to the result concerning SBE reporter in the figure legend.

L277. “The available data suggest” – please point to these data.

Fig 4. The layout of this fig could be improved to make it similar to other figs where the confocal images are followed by fluorescence quantification, thus C after D.

L275-331. I find the presentation of data shown in Fig 4, difficult to follow because the narrative does not follow the order of sub-panels in this fig.

Fig S7 – could the revised model figure be part of the main figs?

Overall, this is a well-conceived and technically sound study that adds valuable information to the field of receptor-mediated cell wall integrity responses, and I would like to congratulate the authors for it.

Reviewer #2 (Comments for the Author):

Proper intracellular responses to cell wall damage or remodeling are important for plants to maintain normal growth and sustain resistance to environmental stresses. Plasma membrane-localized receptor-like kinases play a key role in transducing cell wall signals, among which SUB is an important one that responds to cellulose biosynthesis inhibition. In this study, the authors identified NHL2 and NHL3 as interacting partners of SUB. Genetic data showed that nhl2 and nhl3 mutants exhibited defects in lignin deposition triggered by isoxaben treatment, similar to the sub mutant, suggesting they may function in the same pathway. Further study showed that NHL3 is required for maintaining the abundance of SUB at the plasma membrane. However, the underlying mechanism remains unclear. Overall, this study is relatively preliminary and lacks mechanistic

insights into how SUB and NHL2/3 regulate each other. Additionally, some of the results are not robust enough to support the conclusions. The major points are as follows:

1. In Fig. S3, to confirm the plasma membrane localization of NHL2-GFP and NHL3-GFP, a membrane fractionation assay combined with immunoblotting should be performed.
2. In Fig. 2, the self-activation of NHL3 should be examined in the Y2H assay.
3. Co-IP assays must be performed to validate the interaction between NHL3 and SUB.
4. Fig. 3c does not show the staining images for sub-9, sub-21, nhl2-1, and nhl3-2.
5. Fig. 3c and 3d, how to explain that NHL2 does not interact with SUB, but nhl2-1 single mutant exhibited a similar defect as sub in lignin deposition triggered by isoxaben? What is the relationship between NHL2 and NHL3? How to explain that nhl2-1 nhl3-2 double mutant exhibited a severity of lignin deposition similar to the single mutants?
6. The resolution of the images in Fig. S9 is extremely low.
7. In Fig. 3a, the transcript levels of SUB:EGFP in each genotype should be examined before examining the fluorescence signals. To confirm the reduction of SUB:EGFP at the plasma membrane in nhl3-2 and qky-11, membrane fractionation assays are recommended. Given that nhl2 did not affect the localization of SUB:EGFP to the plasma membrane, how to explain that nhl2 exhibited a similar defect as sub and nhl3 in lignin deposition?
8. In Fig. 4a, the difference in SUB:EGFP localization at the plasma membrane between wild-type, nhl3-2, and qky-11 backgrounds is minimal. Membrane fractionation assays are strongly recommended to validate these results.
9. The authors should investigate the molecular mechanisms by which NHL2 and NHL3 promote the plasma membrane localization of SUB:EGFP.
10. As a kinase, whether SUB phosphorylates NHL2 and NHL3 warrants further investigation.

Reviewer #3 (Comments for the Author):

The article by Boikine et al describe the function of STRUBBELIG (SUB) receptor kinase in the regulation of cellular responses associated to cell wall integrity (CWI) alteration triggered by treatment of plants with the cellulose biosynthesis inhibitors (CBI). The authors discovered that NHL2 and NHL3, two proteins that have been involved previously in plant response to stresses, are additional factors involved in the regulation of CBI response. These findings increase the number of proteins involved in CWI regulation mediated by CIB, with SUB receptor kinase, preciously described by the group, playing a key role. Authors, also tested the genetic and cellular interactions between these SUB and NHL2/NHL3 components. The article data show that the function NHL3 on cell wall integrity control is dependent of SUB, and the article also contains novel sets of biochemically and functionally distinct SUB complexes involved in regulating cell wall integrity and development. The microscopic and genetic analyses performed are of great quality and the characterization of the different complexes identified at the membrane/cellular levels are well documented. The article contains interesting and novel information and a very well characterization of SUB interaction with and NHL3 at the cellular level. However, some of the conclusions of the article are not supported by the data. Also, a more detailed characterization of the functional mechanism of SUB/NHL3 and NHL2 to explain CBI triggered cell wall integrity alterations would be needed. Last, a clear link between cell wall integrity modification and the activation of SUB/NHL3 and NHL2 has not been shown.

Comments
1. The phenotypic determination of alteration of CWI by CBI in the article is just based on the measurement of lignin accumulation in the transition/elongation zone of the root tip and callose production in the cotyledons, that are clearly two molecules that are deposited in the cell walls. However, additional analyses of cell wall integrity alterations (biochemical determination, immunodetection of changes in specific cell wall components (e.g. pectin), etc.) were not performed. A more detailed characterization of cell wall integrity alterations would be needed to distinguish, for example, among NHL2 and NHL3 (dependent on SUB) mediated mechanisms observed and to further support some of the conclusion of the article (e.g. pectin changes, see point 6). Though some data were generated with mutants defective in CESAs required for primary cell wall biosynthesis, these analyses are based on genetics.
2. The article nicely shows that the read-out of CWI alteration triggered by CIB require NHL2 and NHL3, based on the analyses of different set of mutants (single and higher order) since lignin dose not accumulate in nhl mutants. Interestingly, overexpression of SUB rescues the nhl3-2 mutant phenotype. Also. it is shown that CBI disrupts the physical interaction of SUB with NHL3 and QKY. However, the impact of all these interaction disruptions at the regulatory level (downstream signalling, regulation of genes, etc.) have not been determined. For example, what are the differences at the regulatory level between NHL2 and NHL3 activation of CWI mediated responses? Molecular bases of these different mechanisms should be determined, since the readout (lignin accumulation) is identical, but the pathways seem to be different.
3. It has not been characterised how CBI mediates SUB internalization or blocks SUB homo-oligomerization. What is the signal for SUB internalization?
4. Authors suggest that NHL3 is a more general stress response factor than previously believed. NHL3 has been previously shown to participate in cell wall remodelling induced by the perception of pathogens. Whether the mechanism regulated by NHL3 in pathogen perception and CBI-induced cell wall damage regulation share some components or are specific processes has not been determined.
5. Authors suggest that application of isoxaben or thaxtomin A first triggers a likely cell wall-derived signal that rapidly activates SUB signalling, but not very well know cell wall derived DAMPs have been tested experimentally in the experiments performed.
6. Authors propose that CBI-induced changes in the SUB/QKY complex architecture are due to cell wall signalling responding to such secondary alterations in the cell wall, possibly related to pectin, but changes in pectin composition/structure in cell walls upon CIB have not been tested/determined. Can be the phenotypes observed in sub and hhl3 mutants mimicked by

adding exogenously OGs or cellulose-derived DAMPs?
7. NHL2 and NHL3 are not involved in SUB-dependent plant development. Explanation for this?. Is SUB involve in disease resistance?

********************END********************

Version 1:

Reviewer comments:

Reviewer #1

(Comments for the Author)
The Authors have substantially revised the manuscript and responded thoughtfully to most of the concerns raised in the first round. The removal of all NHL2 data simplifies and strengthens the narrative considerably, and several new experiments add mechanistic value.

The concern about the ternary complex (SUB–NHL3–QKY) has been resolved. The Authors have removed this speculation from both the Discussion and the model figure (now Extended Data Fig. 8), focusing instead on the two well-supported binary complexes. The revised model is more parsimonious and better aligned with the available biochemical data. Yet, it would be advisable to discuss more in depth the results concerning NHL3 and QKY localization presented in Extended Data Fig 3b. It seems that the images show co-localization of QKY and NHL3 at the plasma membrane at a resolution of confocal microscope, whereas the lifetime analysis does not support the physical interaction between them. This result is important for better understanding the relationship between NHL3 and QKY.

The concern about the early SUB internalization yet late SUB-dependent lignification has been addressed convincingly. The new data from chc2-1 and chc2-2 mutants (Extended Data Fig. 7), which show hyperlignification mirroring SUB overexpression, provide strong support for the conclusion that SUB functions at the plasma membrane rather than at endosomes.

The concern about the marked kinetic difference between SUB/NHL3 and SUB/QKY dissociation has been reasonably addressed. The new propidium iodide data (Extended Data Fig. 5j,k) provide a plausible cell-biological correlate for the delayed SUB/QKY disruption, and the Authors are appropriately cautious in framing the two-signal model as a hypothesis. This is a reasonable position given the scope of the work.

Moreover, the Authors successfully addressed all queries of Reviewer 2 and Reviewer 3, either in the rebuttal letter or by additional experiments and deletion of data concerning NHL2.

There remain a few minor things to address:

Extended Data Fig 1e and 1f measures potential interaction of NHL3 and plasma membrane labeled by a styryl dye FM4-64. The figure legend wrongly (?) describes the interaction as an interaction between proteins in 1e and 1f.

L. 181-182 and all revised (highlighted) text. Please adjust the format of citations.

L. 200-204. Assuming that the cross OE::SUB 03 with nhl3-2 mutant was analyzed as a hemizygous stock., the results could be interpreted as effect of gene dosage of SUB rather than the need for NHL3. If the stock was homozygotic, then the interpretation is correct. Please add the information on zygosity for the proper interpretation of the results.

L219-226. Please indicate if the tests were carried out in the dark or in the light and address the implications of the light conditions on the results.

Fig. 4 legend. Please add information about the oligomerization experiment to the figure title. Please define “SSE” at the first mention in (a,b).

Extended data Fig. 5k. The authors mention a five hours treatment in the main text (line 263) while the figure shows a six hours label.

The legend for the extended data Fig. 5 (in bold font) misses the information that the figure includes experiments testing the induction of callose by NHL3.

L. 652 “analyzation”

L. 1034 and 1039. “Sidek’s multiple comparison test“

Overall, this is a well-argued study that makes a meaningful contribution to our understanding of receptor-mediated cell wall integrity signalling in plants.

Laura Bacete Cano and Ewa Mellerowicz, Umeå Plant Science Centre, Sweden

Reviewer #2

(Comments for the Author)
The authors only partially addressed my concerns, and overall this study still lacks strong mechanistic insights. Below are some major concerns.

1. In Extended Data Fig. 1, the authors identified SUB-NHL3 using an IP-MS assay. It is recommended that they perform an independent Co-IP assay using double-labeled transgenic plants to confirm this interaction.
2. The authors found that the transcript level of SUB expression was reduced in the nhl3 mutant. How, then, can the authors rule out the possibility that the reduced SUB:EGFP at the plasma membrane of the nhl3 mutant is not merely due to reduced gene expression?
3. How does NHL3 maintain SUB localization at the plasma membrane, and what is the biological significance of CBI-induced SUB internalization?

Reviewer #3

(Comments for the Author)
This revised version of the manuscript is a clear improvement of the previously submitted manuscript. The authors have addressed some of the main issues raised by the reviewer: the demonstration of cell wall alterations upon isoxaben treatment (determination of modification of pectins) and characterization of the role of SUB in disease resistance, tested in the interaction Arabidopsis/powdery mildew. These data partially support the main conclusion of the article. However, the putative response of sub and nhl3 mutants to cell wall derived signals has not been determined, though some hypothesis and conclusions related to this issue have been weakened.

Minor points:
Delete #codes from References:
(Fotin et al., 2004, #48229). In Arabidopsis, CHCs are 328 encoded by two likely redundant genes, CLATHRIN HEAVY CHAIN (CHC) 1 and 329 CHC2 (Scheele & Holstein, 2002; #83464)
Furthermore, since SUB homo-oligomerization depends on QKY (Chen et al., 2023, 440 #104900),

Decision Letter:

Our ref: NPLANTS-251021164A

13th May 2026

Dear Dr. Schneitz,

Thank you for submitting your revised manuscript "A STRUBBELIG-NHL3 cell surface receptor complex mediates the response to cellulose deficiency in Arabidopsis" (NPLANTS-251021164A). It has now been seen by the original referees and their comments are below. The reviewers find that the paper has improved in revision, and therefore we'll be happy in principle to publish it in Nature Plants, pending minor revisions to satisfy the referees' final requests and to comply with our editorial and formatting guidelines. If we think that the final concerns have been sufficiently addressed, we will not send the manuscript back to reviewers.

We are now performing detailed checks on your paper and will send you a checklist detailing our editorial and formatting requirements in about a week. Please do not upload the final materials and make any revisions until you receive this additional information from us.

Thank you again for your interest in Nature Plants Please do not hesitate to contact me if you have any questions.

Sincerely,

Reviewer #1 (Comments for the Author):

The Authors have substantially revised the manuscript and responded thoughtfully to most of the concerns raised in the first round. The removal of all NHL2 data simplifies and strengthens the narrative considerably, and several new experiments add

mechanistic value.

The concern about the ternary complex (SUB–NHL3–QKY) has been resolved. The Authors have removed this speculation from both the Discussion and the model figure (now Extended Data Fig. 8), focusing instead on the two well-supported binary complexes. The revised model is more parsimonious and better aligned with the available biochemical data. Yet, it would be advisable to discuss more in depth the results concerning NHL3 and QKY localization presented in Extended Data Fig 3b. It seems that the images show co-localization of QKY and NHL3 at the plasma membrane at a resolution of confocal microscope, whereas the lifetime analysis does not support the physical interaction between them. This result is important for better understanding the relationship between NHL3 and QKY.

The concern about the early SUB internalization yet late SUB-dependent lignification has been addressed convincingly. The new data from chc2-1 and chc2-2 mutants (Extended Data Fig. 7), which show hyperlignification mirroring SUB overexpression, provide strong support for the conclusion that SUB functions at the plasma membrane rather than at endosomes.

The concern about the marked kinetic difference between SUB/NHL3 and SUB/QKY dissociation has been reasonably addressed. The new propidium iodide data (Extended Data Fig. 5j,k) provide a plausible cell-biological correlate for the delayed SUB/QKY disruption, and the Authors are appropriately cautious in framing the two-signal model as a hypothesis. This is a reasonable position given the scope of the work.

Moreover, the Authors successfully addressed all queries of Reviewer 2 and Reviewer 3, either in the rebuttal letter or by additional experiments and deletion of data concerning NHL2.

There remain a few minor things to address:

Extended Data Fig 1e and 1f measures potential interaction of NHL3 and plasma membrane labeled by a styryl dye FM4-64. The figure legend wrongly (?) describes the interaction as an interaction between proteins in 1e and 1f.

L. 181-182 and all revised (highlighted) text. Please adjust the format of citations.

L. 200-204. Assuming that the cross OE::SUB 03 with nhl3-2 mutant was analyzed as a hemizygous stock., the results could be interpreted as effect of gene dosage of SUB rather than the need for NHL3. If the stock was homozygotic, then the interpretation is correct. Please add the information on zygosity for the proper interpretation of the results.

L219-226. Please indicate if the tests were carried out in the dark or in the light and address the implications of the light conditions on the results.

Fig. 4 legend. Please add information about the oligomerization experiment to the figure title. Please define “SSE” at the first mention in (a,b).

Extended data Fig. 5k. The authors mention a five hours treatment in the main text (line 263) while the figure shows a six hours label.

The legend for the extended data Fig. 5 (in bold font) misses the information that the figure includes experiments testing the induction of callose by NHL3.

L. 652 “analyzation”

L. 1034 and 1039. “Sidek’s multiple comparison test“

Overall, this is a well-argued study that makes a meaningful contribution to our understanding of receptor-mediated cell wall integrity signalling in plants.

Laura Bacete Cano and Ewa Mellerowicz, Umeå Plant Science Centre, Sweden

Reviewer #2 (Comments for the Author):

The authors only partially addressed my concerns, and overall this study still lacks strong mechanistic insights. Below are some major concerns.

1. In Extended Data Fig. 1, the authors identified SUB-NHL3 using an IP-MS assay. It is recommended that they perform an independent Co-IP assay using double-labeled transgenic plants to confirm this interaction.
2. The authors found that the transcript level of SUB expression was reduced in the nhl3 mutant. How, then, can the authors rule out the possibility that the reduced SUB:EGFP at the plasma membrane of the nhl3 mutant is not merely due to reduced gene expression?
3. How does NHL3 maintain SUB localization at the plasma membrane, and what is the biological significance of CBI-induced SUB internalization?

Reviewer #3 (Comments for the Author):

This revised version of the manuscript is a clear improvement of the previously submitted manuscript. The authors have addressed some of the main issues raised by the reviewer: the demonstration of cell wall alterations upon isoxaben treatment (determination of modification of pectins) and characterization of the role of SUB in disease resistance, tested in the interaction Arabidopsis/powdery mildew. These data partially support the main conclusion of the article. However, the putative response of sub and nhl3 mutants to cell wall derived signals has not been determined, though some hypothesis and conclusions related to this issue have been weakened.

Minor points:
Delete #codes from References:
(Fotin et al., 2004, #48229). In Arabidopsis, CHCs are 328 encoded by two likely redundant genes, CLATHRIN HEAVY CHAIN (CHC) 1 and 329 CHC2 (Scheele & Holstein, 2002; #83464)
Furthermore, since SUB homo-oligomerization depends on QKY (Chen et al., 2023, 440 #104900),

Version 2:

Decision Letter:

22nd June 2026

Dear Professor Schneitz,

We are pleased to inform you that your Article entitled "A STRUBBELIG-NHL3 cell surface receptor complex mediates the response to cellulose deficiency in Arabidopsis", has now been accepted for publication in Nature Plants.

Over the next few weeks, your paper will be copyedited to ensure that it conforms to Nature Plants style. We look particularly carefully at the titles of all papers to ensure that they are relatively brief and understandable.

Once your paper is typeset, you will receive an email with a link to choose the appropriate publishing options for your paper and our Author Services team will be in touch regarding any additional information that may be required.

After the grant of rights is completed, you will receive a link to your electronic proof via email with a request to make any corrections within 48 hours. If, when you receive your proof, you cannot meet this deadline, please inform us at rjsproduction@springernature.com immediately.

You will not receive your proofs until the publishing agreement has been received through our system.

You may wish to make your media relations office aware of your accepted publication, in case they consider it appropriate to organize some internal or external publicity. Once your paper has been scheduled you will receive an email confirming the publication details. This is normally 3-4 working days in advance of publication. If you need additional notice of the date and time of publication, please let the production team know when you receive the proof of your article to ensure there is sufficient time to coordinate. Further information on our embargo policies can be found here:
https://www.nature.com/authors/policies/embargo.html

Due to the importance of these deadlines, we ask you please us know now whether you will be difficult to contact over the next month. If this is the case, we ask you provide us with the contact information (email, phone and fax) of someone who will be able to check the proofs on your behalf, and who will be available to address any last-minute problems.

Acceptance of your manuscript is conditional on all authors' agreement with our publication policies (see http://www.nature.com/authors/policies/index.html). In particular your manuscript must not be published elsewhere.

If you have any questions about our publishing options, costs, Open Access requirements, or our legal forms, please contact ASJournals@springernature.com

Authors may need to take specific actions to achieve compliance with funder and institutional open access mandates. If your research is supported by a funder that requires immediate open access (e.g. according to Plan S principles or the NIH public access policy) then you should select the gold OA route, and we will direct you to the compliant route where possible. Because authors warrant under our subscription licensing terms that they haven’t committed to licensing any version of their article under a licence

inconsistent with the terms of our agreement – including the applicable embargo period – publication under the subscription model isn’t suitable for authors whose funders require no embargo.

An online order form for reprints of your paper is available at https://www.nature.com/reprints/author-reprints.html. All co-authors, authors' institutions and authors' funding agencies can order reprints using the form appropriate to their geographical region.

We welcome the submission of potential cover material (including a short caption of around 40 words) related to your manuscript; suggestions should be sent to Nature Plants as electronic files (the image should be 300 dpi at 210 x 297 mm in either TIFF or JPEG format). Please note that such pictures should be selected more for their aesthetic appeal than for their scientific content, and that colour images work better than black and white or grayscale images. Please include a written description of your image and how it was created along with your image files. Please do not try to design a cover with the Nature Plants logo etc., and please do not submit composites of images related to your work. I am sure you will understand that we cannot make any promise as to whether any of your suggestions might be selected for the cover of the journal.

You can now use a single sign-on for all your accounts, view the status of all your manuscript submissions and reviews, access usage statistics for your published articles and download a record of your refereeing activity for the Nature journals.

To assist our authors in disseminating their research to the broader community, our SharedIt initiative provides you with a unique shareable link that will allow anyone (with or without a subscription) to read the published article. Recipients of the link with a subscription will also be able to download and print the PDF.

As soon as your article is published, you will receive an automated email with your shareable link.

With kind regards,

P.S. Click on the following link if you would like to recommend Nature Plants to your librarian
http://www.nature.com/subscriptions/recommend.html#forms

** Visit the Springer Nature Editorial and Publishing website at www.springernature.com/editorial-and-publishing-jobs for more information about our career opportunities. If you have any questions please click here.**

Open Access This Peer Review File is licensed under a Creative Commons Attribution 4.0 International License, which permits use, sharing, adaptation, distribution and reproduction in any medium or format, as long as you give appropriate credit to the original author(s) and the source, provide a link to the Creative Commons license, and indicate if changes were made. In cases where reviewers are anonymous, credit should be given to 'Anonymous Referee' and the source.
The images or other third party material in this Peer Review File are included in the article’s Creative Commons license,

unless indicated otherwise in a credit line to the material. If material is not included in the article's Creative Commons license and your intended use is not permitted by statutory regulation or exceeds the permitted use, you will need to obtain permission directly from the copyright holder.
To view a copy of this license, visit https://creativecommons.org/licenses/by/4.0/

Response to referees:

Reviewer #1 (Comments for the Author):
Boikine et al. show in this manuscript how the recently established new player in cell wall integrity stress, the receptor kinase STRUBBELIG (SUB), could mediate the reaction to cellulose biosynthesis inhibition (CBI). The authors identify NHL3 as a direct interactor of SUB and show their joint role in the Arabidopsis response to CBI. The authors propose that SUB acts as a co-receptor forming several complexes (SUB–NHL3, SUB–NHL3–QKY, and SUB–QKY) that regulate distinct aspects of the CBI response or different developmental responses. The data demonstrate a clear physical interaction between SUB and NHL3, and functional assays show that both are required for normal callose and lignin deposition following cellulose perturbation. Very interestingly, the authors also propose a new time frame for some responses to CBI, such as SUB internalization, which begins within 30 minutes rather than 4–5 hours as previously thought. The study is technically competent and addresses a relevant topic in cell-wall integrity signalling. However, I have some concerns regarding the proposed mechanistic model and interpretation of key findings:

1. Evidence for a ternary complex and its biochemical basis. In the discussion (lines 400–408) the authors propose three distinct SUB-containing complexes, including complex B (SUB–NHL3–QKY) that would specifically regulate lignification. However, the biochemical support for this ternary complex is lacking. NHL3 and QKY cannot directly interact (NHL3–QKY interaction is negative, Supplementary Fig. 4, lines 142–145). Additionally, NHL3, unlike QKY, does not promote SUB homo-oligomerization (lines 271–272: "minimal difference in FA values indicates that NHL3...does not promote SUB homo-multimerization"). These observations suggest that NHL3 and QKY have fundamentally different biochemical roles. The existence of a ternary complex is therefore inferred from overlapping phenotypes, not shown biochemically. The authors acknowledge that NHL3 and QKY function in parallel (lines 284– 285) and propose that "additional SUB complexes containing NHL3 or QKY that are not subject to CBI-induced internalization are likely also present" (lines 412–414). This raises a critical question: if NHL3 and QKY have distinct oligomerization properties and cannot directly interact, what is the molecular basis for postulating they assemble into

the same ternary complex? The model would be stronger if the authors were more explicit about whether complex B represents a simultaneous trimeric assembly or a temporal succession of binary complexes on the same receptor, as stated in the Abstract. The model shown in fig S7 should be revised accordingly and more emphasis should be given to complexes A and C which are well-supported.

We previously mentioned the ternary complex (NHL3-SUB-QKY) because it is a formal possibility due to the shared role in CBI-induced lignification. However, we understand this referee's concerns. Given the lack of experimental evidence for its existence, we removed this section from the Discussion and focused on the two complexes with supporting evidence: SUB/NHL3 and SUB/QKY. In this case, we believe the simplest explanation is as follows: SUB/NHL3 and SUB/QKY are distinct complexes. Both NHL3 and QKY are involved in maintaining SUB at the plasma membrane. The CBI response successively interferes with the architecture of the two complexes by acting on SUB-NHL3 before SUB-QKY (see also below). We have updated the Discussion and the model cartoon.

2. Interpretations of some interactions could be improved from the mechanistic point of view. For example, the temporal dissociation of SUB interactions with differential kinetics. The authors report that isoxaben rapidly dissociates SUB–NHL3 interaction (undetectable by 30 minutes, lines 322–323), while SUB–QKY interaction remains prominent at 30 minutes (line 325) and only shows substantial attenuation after 5 hours (lines 326–330). This marked temporal difference (15 min vs. 5 hours, ~200-fold difference in timescale) is striking and raises an important mechanistic question: what explains this dramatic difference in the kinetics of dissociation? The paper shows that both interactions are disrupted by CBI, but the molecular basis for this temporal hierarchy is not explained. The authors propose (lines 356–357) that "a likely cell-wall-derived signal" rapidly activates SUB, but provide no mechanistic explanation for why this signal would release NHL3 rapidly while leaving QKY relatively stable for hours.

We agree with this reviewer. We cannot provide mechanistic insight into the signal side of the SUB-dependent CBI response. The identification of the CBI-induced signals that are perceived by the two complexes is beyond the scope of this paper. Since we don't know the nature of the signals, we are more neutral when mentioning them. In the revised manuscript we no longer refer to them as "cell wall-derived signals“ but simply as “signals”.

Currently available evidence suggests that CBI induces a complex response involving ROS production, hormone effects, cell wall remodeling (lignin, callose) and so on (Hamann et.al 2009, Engelsdorf et.al. 2019 and other references). It also causes developmental defects, particularly altered root hair patterning and ovule morphogenesis (Chaudhary et.al. 2021 Development 148:dev199425). These developmental defects were found to be caused by the CBI-induced downregulation of SUB activity. They could be rescued by artificially upregulating SUB indicating that SUB downregulation is the main effect of CBI on these developmental processes (Chaudhary et.al. 2021 Development 148:dev199425). We now clarify this in the Introduction.

The SUB/NHL3 and SUB/QKY complexes clearly have different functions. Available data suggest that the SUB/NHL3 complex is necessary for most if not all of the SUB-dependent compensatory CBI response in seedlings (ROS, *SUB* transcript levels, lignin, and callose). In

contrast, since *qky* mutants exhibit an identical phenotype to *sub* mutants with regard to root hair patterning and ovule morphogenesis, the SUB/QKY complex primarily mediates the developmental role of SUB and plays a minor role in the compensatory CBI response, specifically with regard to lignin. Therefore, while it is formally possible, it is probably unlikely that the two complexes respond to the same signal.

Since the CBI-induced disruption of the SUB/NHL3 complex happens rapidly within minutes, we postulate that this is an early response. In contrast, the subsequent disruption of the SUB/QKY complex at around 5 hours of isoxaben exposure appears to be a later consequence of CBI. In support of this notion, we added new evidence revealing that 30 minutes of isoxaben treatment does not obviously affect the propidium iodide-stained part of the cell wall. Propidium iodide labels de-methylesterified pectin (Rounds et.al. 2011 Plant Physiol 157:175). After five hours, however, there is a noticeable increase in cellular uptake of negatively charged cell wall components, likely de-methylesterified pectin fragments, as suggested by the increase in internalized cytoplasmic puncta marked with propidium iodide (see new Extended Data Figure 5j,k). This indicates that CBI eventually affects pectin as well.

Thus, we now propose the existence of at least two different CBI-related signals that differentially affect the SUB/NHL3 and SUB/QKY complexes: an early acting signal for the SUB/NHL3 complex and a different later acting signal for the SUB/QKY complex. The activity of the second, later-acting signal indirectly depends on the earlier activity of the SUB/NHL3 complex. CBI-induced lignification is a late process as the onset of the lignin response begins around 4-6 hours after the onset of CBI (see for example Hamann et.al. 2009 Plant Journal 57:1015). Interestingly, the SUB/QKY complex disruption takes place at about 5 hours of isoxaben treatment. Our data are consistent with the idea that a significant change to the cell wall occurs around five hours of isoxaben exposure generating a second signal that activates the SUB/QKY complex and induces lignification of the root tip.

We believe that the reduction in SUB levels at the plasma membrane caused by the disruption of the SUB/QKY complex induced by CBI dampens the SUB-dependent CBI response, thereby preventing hyperreaction. However, this also results in root hair patterning defects. This is supported by the finding that SUB overexpression rescues the *qky* phenotype (Vaddepalli et al., 2014, Development, 141:4139). Additionally, QKY regulates SUB levels at the plasma membrane without affecting *SUB* transcript levels (Song et al., 2019, Nat. Commun., 10:1744). These developmental defects may be a side effect of CBI, or they may be part of a mechanism that balances stress responses and development. These points are clarified in the Discussion section.

3. The authors report that SUB–NHL3 interaction decreases within 15 minutes of isoxaben treatment (line 322) and SUB internalization increases within 30 minutes (lines 295–297). Yet lignification, a late CBI response measured at 16 hours (lines 164–165), remains SUB-dependent (lines 165–167). This timing raises an important mechanistic question: does SUB signaling require continuous plasma membrane presence, does endosomal localization enhance activation of the pathway (like in the case of BRI1)? Or is SUB is recycled back to the membrane?

As mentioned above CBI-induced lignification starts around 4-6 hours after onset of treatment. We used the 16-hour timepoint to obtain sufficient and reproducible staining. We now outline this point in the paper.

SUB undergoes constitutive internalization followed by degradation (Gao et.al. 2019 J Exp Bot 70:3881; Song et.al. 2019 Nat Comm 10:1744). We have previously shown that this involves constitutive clathrin-mediated endocytosis (CME) (Gao et.al. 2019). With respect to root hair patterning, it was concluded that too much activity of SUB at the plasma membrane interferes with SUB signaling as *chc2* mutants, which show significantly reduced endocytosis rate, exhibited mild root hair patterning defects comparable to overexpression of SUB (Gao et.al. 2019). In the revised version of the manuscript, we investigated isoxaben-induced lignification in two loss-of-function mutants of *CHC2.* We found hyperlignification in the roots in both *chc2* mutants (new Extended Data Figure 7). Since *sub* mutants exhibit reduced lignification and overexpressing *SUB* results in hyperlignification, this finding supports the hypothesis that SUB operates at the plasma membrane in the CBI response. If SUB acted at endosomes, we would expect reduced lignification in *chc2* mutants.

Suggestions listed below mainly address the readability of the paper. Although very clearly written in general, I think some parts of this paper could be improved.

Fig S1a legend – please briefly explain how the protein abundance is measured.

We now provide additional information in the figure legend and in the Co-Immunoprecipitation and Proteomics of the M&M: Proteins bound to anti-GFP nanobodies coupled to agarose beads were analyzed by bottom-up liquid chromatography-mass spectrometry (LC-MS). All displayed protein abundances refer to MaxQuant LFQ intensities which are based on extracted MS1 precursor ion intensities for each identified peptide feature (XIC-based).

Fig S2b and other box-plots figs – please explain what is presented as the box including its horizontal line, possibly in one common sentence for all parts of the figures.

We now state in the statistics section of the M&M: For the box and whisker plots the whisker ends mark the minimum and maximum and the horizontal line the mean of all data. In the supplement we added this line to each relevant figure legend.

Fig S3c,d,e – please briefly explain that these are FRET/FLIM experiments and that the lifetime reduction indicates an interaction at the first mention of this technique. In this fig and other similar figs, please state what represents “n”, number of plants?

We now mention that a reduction in lifetime is indicative of interaction in the main manuscript.

L 91. Please state the full name of LTi6b and indicate if it is an Arabidopsis protein

We now provide the full name and indicate that it is an Arabidopsis protein.

L130 – the part of the sentence directly after “or” – redundant?

No. The sentence highlights the difference between BRI1:GFP vs BRI1:GFP/BAK1mCherry.

L 173. The claim of similar effects in case of callose induction for the different mutants should be narrowed as not all mutants were tested for callose induction as shown in Fig. S7 and qky mutants were previously reported not to affect this response.

We now explicitly mention *sub-21* and *nhl3-2* mutants.

Fig. 3hi – I presume TMO7:GPF constructs were used as a positive control. This should be explained. The identity of TMO7-encoded protein should be stated and the choice of this reporter explained. The attention of the reader should be pointed to the result concerning SBE reporter in the figure legend.

Fixed.

L277. “The available data suggest” – please point to these data.

We now mention Fig. 3 and the two relevant papers (Chaudhary et.al. 2021, Song et.al. 2019).

Fig 4. The layout of this fig could be improved to make it similar to other figs where the confocal images are followed by fluorescence quantification, thus C after D.

Fixed.

L275-331. I find the presentation of data shown in Fig 4, difficult to follow because the narrative does not follow the order of sub-panels in this fig.

We rearranged the figure accordingly.

Fig S7 – could the revised model figure be part of the main figs?

Since we are reaching the length limits we kept the now simplified figure in the supplement (Extended Data Figure 8).

Overall, this is a well-conceived and technically sound study that adds valuable information to the field of receptor- mediated cell wall integrity responses, and I would like to congratulate the authors for it.

Thank you. We appreciate these kind words.

Reviewer #2 (Comments for the Author):
Proper intracellular responses to cell wall damage or remodeling are important for plants to maintain normal growth and sustain resistance to environmental stresses. Plasma membrane-localized receptor-like kinases play a key role in transducing cell wall signals, among which SUB is an important one that responds to cellulose biosynthesis inhibition. In this study, the authors identified NHL2 and NHL3 as interacting partners of SUB. Genetic data showed that nhl2 and nhl3 mutants exhibited defects in lignin deposition triggered by isoxaben treatment, similar to the sub mutant, suggesting they may function in the same pathway. Further study showed that NHL3 is required for maintaining the abundance of SUB at the plasma membrane. However, the underlying mechanism remains unclear. Overall, this study is relatively preliminary and lacks mechanistic insights into how SUB and NHL2/3 regulate each other. Additionally, some of the results are not robust enough to support the conclusions. The major points are as follows:

1. In Fig. S3, to confirm the plasma membrane localization of NHL2-GFP and NHL3-GFP, a membrane fractionation assay combined with immunoblotting should be performed.

We currently do not understand the mechanism how NHL2 functions in the context of the response to CBI. To avoid confusion, we removed all data regarding NHL2 from the manuscript.

The requested experiment has already been performed for NHL3 by several authors. Plasma membrane localization for NHL3 was demonstrated biochemically using membrane fractions/immunoblotting and by immunolocalization (Varet et.al. 2003 Plant Physiol 132:2023-2033). This was confirmed by confocal microscopy using a NHL3-mCherry reporter (Tee et.al. 2023 PNAS 120:e2216397120). Our data provide additional confirmation.

2. In Fig. 2, the self-activation of NHL3 should be examined in the Y2H assay.

We have performed the requested experiment and present it in new Extended Data Fig. 2g. No self-activation of NHL3 could be observed.

3. Co-IP assays must be performed to validate the interaction between NHL3 and SUB.

We do not understand this point. In this paper we identified NHL3 in a co-IP assay in seedlings using SUB:EGFP as bait (Fig.1, Extended Data Fig. 1). We confirmed physical interaction by a Y2H assay and by FRET-FLIM in transgenic Arabidopsis seedlings carrying functional reporters.

4. Fig. 3c does not show the staining images for sub-9, sub-21, nhl2-1, and nhl3-2.

We removed all NHL2 data and rearranged Fig. 3c now showing all the corresponding stained and unstained images.

5. Fig. 3c and 3d, how to explain that NHL2 does not interact with SUB, but nhl2-1 single mutant exhibited a similar defect as sub in lignin deposition triggered by isoxaben? What is the relationship between NHL2 and NHL3? How to explain that nhl2-1 nhl3-2 double mutant exhibited a severity of lignin deposition similar to the single mutants?

We believe this reviewer refers to Fig. 2C,D? As detailed above, we removed all data regarding NHL2 from the manuscript.

6. The resolution of the images in Fig. S9 is extremely low.

Due to the treatment, it is difficult to obtain crisp images of roots that did not stain with phloroglucinol. We have done our best to include better images in the revised version.

7. In Fig. 3a, the transcript levels of SUB:EGFP in each genotype should be examined before examining the fluorescence signals.

It was previously shown that *QKY* does not influence *SUB* expression (Song et.al. 2019 Nat Commun 10:1744). In the revised version we included an investigation of *SUB* expression in *nhl3-2* by qPCR (new Extended Data Fig. 6c). We found a slighly reduced expression indicating that apart from maintaining SUB at the plasma membrane, NHL3 is also required for *SUB* expression. We now highlight and discuss this new finding in the revised paper.

To confirm the reduction of SUB:EGFP at the plasma membrane in nhl3-2 and qky-11, membrane fractionation assays are recommended.

Please see our response to point 8 of this reviewer.

Given that nhl2 did not affect the localization of SUB:EGFP to the plasma membrane, how to explain that nhl2 exhibited a similar defect as sub and nhl3 in lignin deposition?

We removed our data regarding NHL2 from the revised manuscript.

8. In Fig. 4a, the difference in SUB:EGFP localization at the plasma membrane between wild-type, nhl3-2, and qky-11 backgrounds is minimal. Membrane fractionation assays are strongly recommended to validate these results.

We beg to differ. We feel that the reduction is not minimal as documented visually and by quantification of the signal. Moreover, Song et.al. 2019 Nat Commun 10:1744 and Chen et.al. 2023 Plant Phys 193:2538 have already convincingly demonstrated that SUB:EGFP signal at the plasma membrane is noticeably reduced in *qky* mutants.

9. The authors should investigate the molecular mechanisms by which NHL2 and NHL3 promote the plasma membrane localization of SUB:EGFP.

We believe that we have achieved this in our paper on NHL3. Our data reveal that SUB is held in place at the plasma membrane through a physical interaction with NHL3. CBI-based interference with this interaction coincides with the onset of increased SUB internalization. As outlined above, we have removed our data regarding NHL2 from the revised manuscript.

10. As a kinase, whether SUB phosphorylates NHL2 and NHL3 warrants further investigation.

Work from our group (Chevalier et.al. 2005 PNAS 102:9074-9079) and the lab of John Schiefelbein (Kwak et.al. 2014 Plant Physiol 166:976-987) has shown that SUB is an atypical receptor kinase as enzymatic activity of the kinase domain is not required for its function. Thus, we did not explore whether SUB phosphorylates NHL2 or NHL3.

Reviewer #3 (Comments for the Author):

The article by Boikine et al describe the function of STRUBBELIG (SUB) receptor kinase in the regulation of cellular responses associated to cell wall integrity (CWI) alteration triggered by treatment of plants with the cellulose biosynthesis inhibitors (CBI). The authors discovered that NHL2 and NHL3, two proteins that have been involved previously in plant response to stresses, are additional factors involved in the regulation of CBI response. These findings increase the number of proteins involved in CWI regulation mediated by CIB, with SUB receptor kinase, preciously described by the group, playing a key role. Authors, also tested the genetic and cellular interactions between these SUB and NHL2/NHL3 components. The article data show that the function NHL3 on cell wall integrity control is dependent of SUB, and the article also contains novel sets of biochemically and functionally distinct SUB complexes involved in regulating cell wall integrity and development. The microscopic and genetic analyses performed are of great quality and the characterization of the different complexes identified at the membrane/cellular levels are well documented. The article contains interesting and novel information and a very well characterization of SUB interaction with and NHL3 at the cellular level. However, some of the conclusions of the article are not supported by the data. Also, a more detailed characterization of the functional mechanism of SUB/NHL3 and NHL2 to explain CBI triggered cell wall integrity alterations would be needed. Last, a clear link between cell wall integrity modification and the activation of SUB/NHL3 and NHL2 has not been shown.

Comments

1. The phenotypic determination of alteration of CWI by CBI in the article is just based on the measurement of lignin accumulation in the transition/elongation zone of the root tip and callose production in the cotyledons, that are clearly two molecules that are deposited in the cell walls. However, additional analyses of cell wall integrity alterations (biochemical determination, immunodetection of changes in specific cell wall components (e.g. pectin), etc.) were not performed. A more detailed characterization of cell wall integrity alterations would be needed to distinguish, for example, among NHL2 and NHL3 (dependent on SUB) mediated mechanisms observed and to further support some of the conclusion of the article

(e.g. pectin changes, see point 6). Though some data were generated with mutants defective in CESAs required for primary cell wall biosynthesis, these analyses are based on genetics.

Since the role of NHL2 in the CBI process could not be determined more precisely, we eliminated all data about NHL2 from the revised version of the manuscript.

Isoxaben induces a multifaceted response in seedlings. Our focus here is on the molecular details of how the SUB-dependent response to CBI is regulated at the plasma membrane. A detailed description of the many CBI-induced changes in the cell wall warrants its own publication and is beyond the scope of this paper. Nevertheless, we have explored isoxaben-induced changes in the cell wall using propidium iodide as a marker (new Extended Data Fig. 5j,k). Propidium iodide labels de-methylesterified pectin (Rounds et.al. 2011 Plant Physiol 157:175). We could show that after five hours of isoxaben exposure, there is a notable increase in cytoplasmic PI-labelled puncta, likely containing de-methylesterified pectin fragments. This finding suggests that CBI eventually alters the status of the pectin layer of the cell wall in roots of Arabidopsis seedlings.

2. The article nicely shows that the read-out of CWI alteration triggered by CIB require NHL2 and NHL3, based on the analyses of different set of mutants (single and higher order) since lignin dose not accumulate in nhl mutants. Interestingly, overexpression of SUB rescues the nhl3-2 mutant phenotype. Also. it is shown that CBI disrupts the physical interaction of SUB with NHL3 and QKY. However, the impact of all these interaction disruptions at the regulatory level (downstream signalling, regulation of genes, etc.) have not been determined. For example, what are the differences at the regulatory level between NHL2 and NHL3 activation of CWI mediated responses? Molecular bases of these different mechanisms should be determined, since the readout (lignin accumulation) is identical, but the pathways seem to be different.

As outlined above we have removed all data relating to NHL2.

We consider the accumulation of lignin or callose as part of the downstream signaling response which represents a direct read-out for cell wall remodeling. We published earlier that SUB controls early CBI-induced ROS. In the revised version, we tested the early ROS response in *nhl3* mutants as well. As can be seen in new Fig. 2, *sub* and *nhl3* mutants show a similar behavior with a reduced ROS response upon isoxaben treatment starting at around 30 minutes. We also investigated *SUB* expression levels in *nhl3* by qPCR (new Extended Data Fig. 6c). The result indicates that apart from maintaining SUB at the plasma membrane NHL3 is also required for *SUB* expression levels. QKY does not affect *SUB* mRNA levels (Song et.al. 2019 Nat Commun 10:1744). Thus, we now offer insight into several further downstream responses of NHL3 activity, including the regulation of early ROS accumulation, *SUB* expression levels, and cell wall remodeling (e.g., lignin and callose).

3. It has not been characterised how CBI mediates SUB internalization or blocks SUB homo-oligomerization.

We do not understand this point. In the paper, we reveal that CBI attenuates NHL3's physical binding to SUB within 15 minutes. This coincides with the beginning of SUB internalization. In

addition, in a *nhl3* mutant, there is less SUB at the plasma membrane and more SUB-containing internal puncta. Furthermore, overexpressing SUB restores the *nhl3-2* defect in CBI-induced lignification. This finding suggests that artificially increasing SUB levels bypasses the need for NHL3 to maintain them at the plasma membrane. It also suggests that NHL3 acts through SUB in the CBI response.

Previous work by us and others has shown that QKY physically interacts with SUB and maintains it at the plasma membrane (e.g., Vaddepalli et.al. 2014 Development 141:4139; Song et al., 2019, Nat. Commun., 10:1744; Chen et al., 2023, Plant Physiology, 193:2538). Thus, we outline a mechanism by which CBI affects SUB internalization: first by interfering with the SUB/NHL3 complex, and then, hours later, with the SUB/QKY complex.

Regarding the question of how CBI blocks SUB homooligomerization, the following explanation is offered: SUB homooligomerization is QKY-dependent (Chen et al., 2023, Plant Physiology, 193:2538). We propose that CBI attenuates this process by interfering with the SUB/QKY complex. This notion is now mentioned in the Discussion section of the revised version.

What is the signal for SUB internalization?

The onset of CBI rapidly induces SUB internalization. We do not know the molecular players involved, such as the ligand(s). Identifying the ligand, for example, is beyond the scope of this paper. See also our response to point 2 by referee 1.

4. Authors suggest that NHL3 is a more general stress response factor than previously believed. NHL3 has been previously shown to participate in cell wall remodelling induced by the perception of pathogens. Whether the mechanism regulated by NHL3 in pathogen perception and CBI-induced cell wall damage regulation share some components or are specific processes has not been determined.

This paper focuses on SUB-relevant events that occur at the plasma membrane. Our data indicate that NHL3 represents such a shared component as it affects pathogen induced callose deposition (Tee et.al. 2023 PNAS 120:e2216397120) as well as CBI-induced callose (and lignin) deposition (this study). We do not have evidence that SUB plays a role in the pathogen response. We have tested a role of SUB in the response to *Erysiphe cruciferarum* infection However, we found no effect. We included those data in new Extended Data Fig. 4e.

5. Authors suggest that application of isoxaben or thaxtomin A first triggers a likely cell wall-derived signal that rapidly activates SUB signalling, but not very well know cell wall derived DAMPs have been tested experimentally in the experiments performed.

The identification of the specific ligands for the SUB/NHL3 and SUB/QKY complexes are beyond the scope of this paper. Since we don't know the nature of the signals, we now no longer refer to them as "cell wall-derived signals“ but simply as “signals” in the revised manuscript."

It was shown in previous work that the isoxaben-induced CWD response does not involve OG signaling (Engelsdorf et.al. 2018 Sci Signal 11:eaao3070).

In our lab we have performed some experiments exposing seedlings to cellulase, xyloglucanase and pectinase. Although the treatments caused altered cell morphology, they failed to result in lignification of the root tip. These experiments are part of another manuscript which is currently in preparation.

6. Authors propose that CBI-induced changes in the SUB/QKY complex architecture are due to cell wall signalling responding to such secondary alterations in the cell wall, possibly related to pectin, but changes in pectin composition/structure in cell walls upon CIB have not been tested/determined.

Building on the finding that propidium iodide (PI) labels de-methylesterified pectin (Rounds et.al. 2011 Plant Physiol 157:175), we provide new evidence regarding the effects of isoxaben. Short-term treatment (30 minutes) does not visibly affect PI cell wall staining; however, prolonged treatment (five hours) triggers the internalization of PI-marked cytoplasmic puncta (new Extended Data Fig. 5j,k). This suggests the uptake of negatively charged pectin fragments and suggests that CBI-induced stress eventually extends to the pectin matrix.

Although our new data support the notion that CBI-induced cell wall damage also affects the status of cell wall pectin we do not demonstrate that pectin relates to a SUB relevant signal. Thus, we removed "possibly related to pectin” from the sentence.

Can be the phenotypes observed in sub and hhl3 mutants mimicked by adding exogenously OGs or cellulose-derived DAMPs?

Since the various enzyme treatments failed to result in lignification, we did not test cellulose-derived DAMPs in such assays. We may also not expect an effect as these elicitors induce plant defense responses (for example Souza et.al. 2017 Plant Physiol 173:2383) and our data do not support a role for SUB in plant defense. In addition, as mentioned above, the isoxaben-induced CWD response does not involve OG signaling.

7. NHL2 and NHL3 are not involved in SUB-dependent plant development. Explanation for this?.

As outlined above we have removed all data relating to NHL2.

Based on its structure and the available results, our current explanation is that SUB functions as a multifunctional co-receptor, similar to for example BAK1, and is a component of several receptor complexes. Due to their different composition, the complexes differ in their function. For example, the published data on SUB and QKY indicate that a SUB/QKY complex is required for development and CBI-induced lignification but not for CBI-induced callose

formation (Vaddepalli et.al. 2014 Development 141:4139; Song et.al. 2019 Nat Comm 10:1744; Chaudhary et.al. 2020 PLoS Genetics 16: e1008433; Chaudhary et.al. 2021 Development 148: dev199425; Chen et.al. 2023 Plant Physiol 193:2538). NHL3 is part of a stress-specific SUB-complex that does not play an obvious role in development. We modified the Discussion to clarify this notion. Please see also our response to point 2 of reviewer 1.

Is SUB involve in disease resistance?

In collaboration with experts in plant immunity, we have performed experiments testing a role for SUB in the response to infection with powdery mildew (*E. cruciferarum*). The results were negative. They are included in the revised manuscript (new Extended Data Fig. 4e).

Reviewer #1 (Comments for the Author):

The Authors have substantially revised the manuscript and responded thoughtfully to most of the concerns raised in the first round. The removal of all NHL2 data simplifies and strengthens the narrative considerably, and several new experiments add mechanistic value.

The concern about the ternary complex (SUB–NHL3–QKY) has been resolved. The Authors have removed this speculation from both the Discussion and the model figure (now Extended Data Fig. 8), focusing instead on the two well-supported binary complexes. The revised model is more parsimonious and better aligned with the available biochemical data. Yet, it would be advisable to discuss more in depth the results concerning NHL3 and QKY localization presented in Extended Data Fig 3b. It seems that the images show co-localization of QKY and NHL3 at the plasma membrane at a resolution of confocal microscope, whereas the lifetime analysis does not support the physical interaction between them. This result is important for better understanding the relationship between NHL3 and QKY.

The concern about the early SUB internalization yet late SUB-dependent lignification has been addressed convincingly. The new data from chc2-1 and chc2-2 mutants (Extended Data Fig. 7), which show hyperlignification mirroring SUB overexpression, provide strong support for the conclusion that SUB functions at the plasma membrane rather than at endosomes.

The concern about the marked kinetic difference between SUB/NHL3 and SUB/QKY dissociation has been reasonably addressed. The new propidium iodide data (Extended Data Fig. 5j,k) provide a plausible cell-biological correlate for the delayed SUB/QKY disruption, and the Authors are appropriately cautious in framing the two-signal model as a hypothesis. This is a reasonable position given the scope of the work.

Moreover, the Authors successfully addressed all queries of Reviewer 2 and Reviewer 3, either in the rebuttal letter or by additional experiments and deletion of data concerning NHL2.

Thank you for your positive comments.

There remain a few minor things to address:

Extended Data Fig 1e and 1f measures potential interaction of NHL3 and plasma membrane labeled by a styryl dye FM4-64. The figure legend wrongly (?) describes the interaction as an interaction between proteins in 1e and 1f.

We believe these reviewers refer to Extended Data Fig. 2e/f. Indeed the interactions are not between proteins but between the GFP and the FM4-64-labelled plasma membrane. We fixed the error.

L. 181-182 and all revised (highlighted) text. Please adjust the format of citations.

Fixed.

L. 200-204. Assuming that the cross OE::SUB 03 with nhl3-2 mutant was analyzed as a hemizygous stock., the results could be interpreted as effect of gene dosage of SUB rather than the need for NHL3. If the stock was homozygotic, then the interpretation is correct. Please add the information on zygosity for the proper interpretation of the results.

The line is homozygous for the *nhl3-2* mutation and homozygous for the OE::SUB O3 construct. We added clarification to this sentence. It now reads: "…and analyzed the phenotype of a line homozygous for the *nhl3-2* mutation and homozygous for the pUBQ::SUB:mCherry O3 construct (*OE::SUB O3 nhl3-2*)."

L219-226. Please indicate if the tests were carried out in the dark or in the light and address the implications of the light conditions on the results.

The phloroglucinol assays in this paper focused on the root tips (the early elongation zone) and were performed in the light. This region overlaps with the expression pattern of *SUB* in the seedling root. The *prc1-1* roots are shorter when seedlings are grown in the light (Fagard et al., 2000, Plant Cell, 12:2409), but a phloroglucinol signal is undetectable (e.g., Hématy et al., 2007, CB, 17:922; our data). Other *prc1*-related publications employed dark-grown seedlings when analyzing various aspects, including lignification of the hypocotyl, because hypocotyl cells drastically elongate when seedlings are grown in the dark. However, Hématy et al. (2007) also showed that, while the hypocotyl and mature root of dark-grown *prc1-1* seedlings exhibited a phloroglucinol signal, the root tip did not. Thus, a phloroglucinol signal does not appear in the root tip of either light- or dark-grown *prc1* seedlings. Given this scenario and our focus on the root tip, we do not outline the differences between light- and dark-grown seedlings. Nevertheless, we now mention in the text that we assay the phloroglucinol signal in light-grown seedlings and refer to the Hématy 2007 paper.

The two sentences changed to: "In our assay, untreated, light-grown *prc1-1* seedlings failed to exhibit root tip lignification, as evidenced by an absence of detectable phloroglucinol staining, which corroborates prior findings (Hématy et al., 2007). Under these conditions, the reduction in cellulose levels in *prc1-1* is insufficient to induce root tip lignification, which is likely due to the presence of compensating CESA complexes."

Fig. 4 legend. Please add information about the oligomerization experiment to the figure title. Please define "SSE" at the first mention in (a,b).

Fixed. We now mention the oligomerization experiment in the title of the figure legend.

Extended data Fig. 5k. The authors mention a five hours treatment in the main text (line 263) while the figure shows a six hours label.

Fixed in the main text. It was a six-hour treatment. We also mention the isoxaben/PI experiment in the title of the legend.

The legend for the extended data Fig. 5 (in bold font) misses the information that the figure includes experiments testing the induction of callose by NHL3.

Fixed. The legend title now reads: "Comparison of isoxaben-induced phloroglucinol and aniline blue staining in Col-0, *nhl3,* and isoxaben-induced PI internalization."

L. 652 "analyzation"

Fixed.

L. 1034 and 1039. "Sidek's multiple comparison test"

Fixed.

Overall, this is a well-argued study that makes a meaningful contribution to our understanding of receptor-mediated cell wall integrity signalling in plants.

Laura Bacete Cano and Ewa Mellerowicz, Umeå Plant Science Centre, Sweden

Reviewer #2 (Comments for the Author):

The authors only partially addressed my concerns, and overall this study still lacks strong mechanistic insights. Below are some major concerns.

1. In Extended Data Fig. 1, the authors identified SUB-NHL3 using an IP-MS assay. It is recommended that they perform an independent Co-IP assay using double-labeled transgenic plants to confirm this interaction.

We provide three independent lines of evidence: untargeted co-IP in Arabidopsis seedlings using SUB:GFP, provided via a complementing construct, as bait; yeast two-hybrid interactions; and FRET-FLIM in Arabidopsis seedlings in complementing lines (which in our experience is rarely done). In the absence of a SUB antibody, our untargeted co-IP experiment using SUB:GFP is the best we can do, in our opinion.

2. The authors found that the transcript level of SUB expression was reduced in the nhl3 mutant. How, then, can the authors rule out the possibility that the reduced SUB:EGFP at the plasma membrane of the nhl3 mutant is not merely due to reduced gene expression?

We find that the *SUB* transcript levels are reduced by 26 percent in *nhl3* in comparison to wild type while the SUB:EGFP signal at the plasma membrane is reduced by 60 percent. Thus, the mild reduction in transcript levels does not explain the much stronger reduction in SUB:EGFP plasma membrane signal. In addition, the wortmannin treatment (which inhibits internalization of plasma membrane factors among other processes) blocks SUB:EGFP internalization in *nhl3* as well and results in a reporter signal at the plasma membrane comparable to mock-treated wild type (Fig. 4g-i).

3. How does NHL3 maintain SUB localization at the plasma membrane, and what is the biological significance of CBI-induced SUB internalization?

Our data provide evidence for the notion that a physical interaction between NHL3 and SUB keeps SUB at the plasma membrane by attenuating its constitutive internalization. Regarding the biological relevance of CBI-induced SUB internalization, we propose that a CBI-induced signal leads to ligand-induced, receptor-mediated internalization. This is a well-described, general process that usually attenuates signal transduction at the plasma membrane by removing the receptor from the cell surface. It often regulates the amplitude of signaling mediated by the receptor. For clarification, we added a corresponding sentence to the second paragraph of the Discussion.

Reviewer #3 (Comments for the Author):

This revised version of the manuscript is a clear improvement of the previously submitted manuscript. The authors have addressed some of the main issues raised by the reviewer: the demonstration of cell wall alterations upon isoxaben treatment (determination of modification of pectins) and characterization of the role of SUB in disease resistance, tested in the interaction Arabidopsis/powdery mildew. These data partially support the main conclusion of the article. However, the putative response of sub and nhl3 mutants to cell wall derived signals has not been determined, though some hypothesis and conclusions related to this issue have been weakened.

Minor points:
Delete #codes from References:
(Fotin et al., 2004, #48229). In Arabidopsis, CHCs are 328 encoded by two likely redundant genes, CLATHRIN HEAVY CHAIN (CHC) 1 and 329 CHC2 (Scheele & Holstein, 2002; #83464) Furthermore, since SUB homo-oligomerization depends on QKY (Chen et al., 2023, 440 #104900),

Fixed.
